# Mechanism of validamycin A inhibiting DON biosynthesis and synergizing with DMI fungicides against *Fusarium graminearum*

**DOI:** 10.1111/mpp.13060

**Published:** 2021-05-02

**Authors:** Chuanhong Bian, Yabing Duan, Qian Xiu, Jueyu Wang, Xian Tao, Mingguo Zhou

**Affiliations:** ^1^ College of Plant Protection Nanjing Agricultural University Nanjing China

**Keywords:** acid trehalase, deoxynivalenol, DMI fungicides, *Fusarium graminearum*, neutral trehalase, validamycin A

## Abstract

Deoxynivalenol (DON) is a vital virulence factor of *Fusarium graminearum*, which causes Fusarium head blight (FHB). We recently found that validamycin A (VMA), an aminoglycoside antibiotic, can be used to control FHB and inhibit DON contamination, but its molecular mechanism is still unclear. In this study, we found that both neutral and acid trehalase (FgNTH and FgATH) are the targets of VMA in *F*. *graminearum*, and the deficiency of FgNTH and FgATH reduces the sensitivity to VMA by 2.12‐ and 1.79‐fold, respectively, indicating that FgNTH is the main target of VMA. We found FgNTH is responsible for vegetative growth, FgATH is critical to sexual reproduction, and both of them play an important role in conidiation and virulence in *F*. *graminearum*. We found that FgNTH resided in the cytoplasm, affected the localization of FgATH, and positively regulated DON biosynthesis; however, FgATH resided in vacuole and negatively regulated DON biosynthesis. FgNTH interacted with FgPK (pyruvate kinase), a key enzyme in glycolysis, and the interaction was reduced by VMA; the deficiency of FgNTH affected the localization of FgPK under DON induction condition. Strains with a deficiency of FgNTH were more sensitive to demethylation inhibitor (DMI) fungicides. FgNTH regulated the expression level of *FgCYP51A* and *FgCYP51B* by interacting with FgCYP51B. Taken together, VMA inhibits DON biosynthesis by targeting FgNTH and reducing the interaction between FgNTH and FgPK, and synergizes with DMI fungicides against *F*. *graminearum* by decreasing *FgCYP51A* and *FgCYP51B* expression.

## Graphical abstract

VMA inhibits DON biosynthesis by reducing the interaction between FgNTH and FgPK, and synergizes with demethylation inhibitor fungicides against *Fusarium graminearum* via the interaction between FgNTH with FgCYP51B.

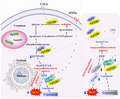

## 1 INTRODUCTION

*Fusarium graminearum* is the most common causal agent of Fusarium head blight (FHB), a destructive disease of wheat in many parts of the world (Jiang et al., 2020; Osborne & Stein, 2007; Snijders, 1990). *F*. *graminearum* is also capable of producing trichothecene mycotoxins in crops, which contributes to the virulence of the fungus and presents a serious threat to food safety and human healthy (Duan et al., 2020; Goswami & Kistler, 2004; Rocha et al., 2005).

The type B trichothecene deoxynivalenol (DON) is the most prevalent toxin associated with FHB (Foroud & Eudes, 2009; Tang et al., 2018). The DON biosynthesis pathway in *F*. *graminearum* has been well described (Brown et al., 2004; Kimura et al., 2007). Farnesyl pyrophosphate (FPP), the precursor of DON biosynthesis, is converted from acetyl‐CoA via the isoprenoid pathway, and acetyl‐CoA is converted from pyruvate by pyruvate dehydrogenase (Zhang et al., 2016). DON production is also regulated by some key enzymes involved in glycolysis, the trehalose pathways, and the tricarboxylic acid (TCA) cycle, such as hexokinase, trehalose‐6‐phosphate phosphatase, and isocitrate dehydrogenase (Li et al., 2019; Song et al., 2014; Zhang et al., 2016; Zhou et al., 2020).

FHB has been commonly controlled by application of fungicides, including benzimidazoles (carbendazim, benomyl, and thiabendazole) and sterol demethylation inhibitors (DMIs; tebuconazole, propiconazole, and prothioconazole) (Duan et al., 2019; McMullen et al., 2012; Yuan & Zhou, 2005). However, the overuse of carbendazim has resulted in the appearance of many carbendazim‐resistant *Fusarium* populations in China (Duan et al., 2014), and a tebuconazole‐resistant isolate of *F*. *graminearum* was also discovered in the United States (Spolti et al., 2014). In addition to being difficult to control, carbendazim‐resistant *Fusarium* populations produce more DON than nonresistant populations (Qiu & Shi, 2014; Zhang et al., 2016). It follows that the control of FHB epidemic and DON production in grain remains a major challenge (Dweba et al., 2017; Wegulo et al., 2015).

We previously reported that, when applied with DMI fungicides, validamycin A (VMA) has synergistic effects in terms of decreasing FHB and mycotoxin content in wheat (Li et al., 2019). VMA is an antibiotic that is produced by *Streptomyces hygroscopicus* var. *limoneus* (Iwasa et al., 1970); it is also named jinggangmycin A when extracted from *S*. *hygroscopicus* var. *jinggangensis* (Shen, 1981). VMA has been used to control rice sheath blight caused by *Rhizoctonia solani* for over 50 years in China, and no VMA‐resistant isolates have been reported in the field (Zhou et al., 1991; Chen et al., 2010). VMA causes abnormal branching of hyphae; however, because the hyphae of *R*. *solani* are multinucleate, it is very difficult to study the molecular mechanism of VMA on *R*. *solani* via genetic transformation (Müller et al., 1995; Robson et al., 1989, 1991; Shigemoto, 1992). VMA can induce broad‐spectrum resistance involving salicylic acid and jasmonic acid/ethylene signalling pathways in plants (Bian et al., 2020). Sterol synthesis inhibitors (azole drugs) have been applied to control FHB for more than 30 years, leading to resistance increasing in some pathogenic fungi, including *Mycosphaerella graminicola*, *Blumeria graminis*, *Aspergillus fumigatus*, and *Candida albicans* (Becher et al., 2010; Godet & Limpert, 1998; Liu et al., 2019), and a tebuconazole‐resistant isolate of *F*. *graminearum* has been discovered in the United States (Spolti et al., 2014). Reducing the usage of fungicides to delay the development of resistance is therefore urgently needed.

Trehalases, which hydrolyse trehalose into two molecules of glucose, are classically divided into two types according to their optimal pH: acid trehalases (ATH), which have an acidic pH optimum and high heat stability, and neutral trehalases (NTH), which have a neutral pH optimum and low heat stability (Bonini et al., 2004). Trehalose (α‐d‐glucopyranosyl‐(1→1)‐α‐d‐glucopyranoside) is a nonreducing disaccharide found in many organisms such as bacteria, fungi, invertebrates, and plants (Avonce et al., 2005; Paul et al., 2008). As a consequence, trehalase inhibitors might function as new pesticides. The absence of trehalose in *F*. *graminearum* caused a significant reduction in development, virulence, and mycotoxin production (Song et al., 2014). Although VMA is recognized as a potent trehalase inhibitor, it is unclear whether it targets NTH, ATH, or both. Our laboratory previously reported that VMA decreases pyruvate and acetyl‐CoA contents and reduces DON production by inhibiting the activity of NTH in *F*. *graminearum* (Li et al., 2019). However, whether ATH is also the target of VMA in *F*. *graminearum* is unclear. The functions of neutral and acid trehalases and the mechanism by which trehalase regulates DON biosynthesis in *F*. *graminearum* are also unclear.

The aim of this study is to address this knowledge gap, and reveal the targets of VMA at gene level and its molecular mechanism of decreasing DON biosynthesis and synergizing with DMIs fungicides against FHB. In this study, we confirmed that the targets of VMA are FgNTH and FgATH, and revealed that VMA decreases DON biosynthesis by reducing the interaction between FgNTH and FgPK. We demonstrated that VMA has synergistic effect with DMIs fungicides through FgNTH positively regulating the expression level of *FgCYP51A* and *FgCYP51B* by interacting with FgCYP51B in *F*. *graminearum*. The results of this study will be helpful to guide the practical application of VMA and reduce the dose of DMI fungicides for control of FHB and DON contamination in wheat.

## 2 RESULTS

### 2.1 Identification of the neutral and acid trehalases in *F. graminearum*

**FIGURE 1:**
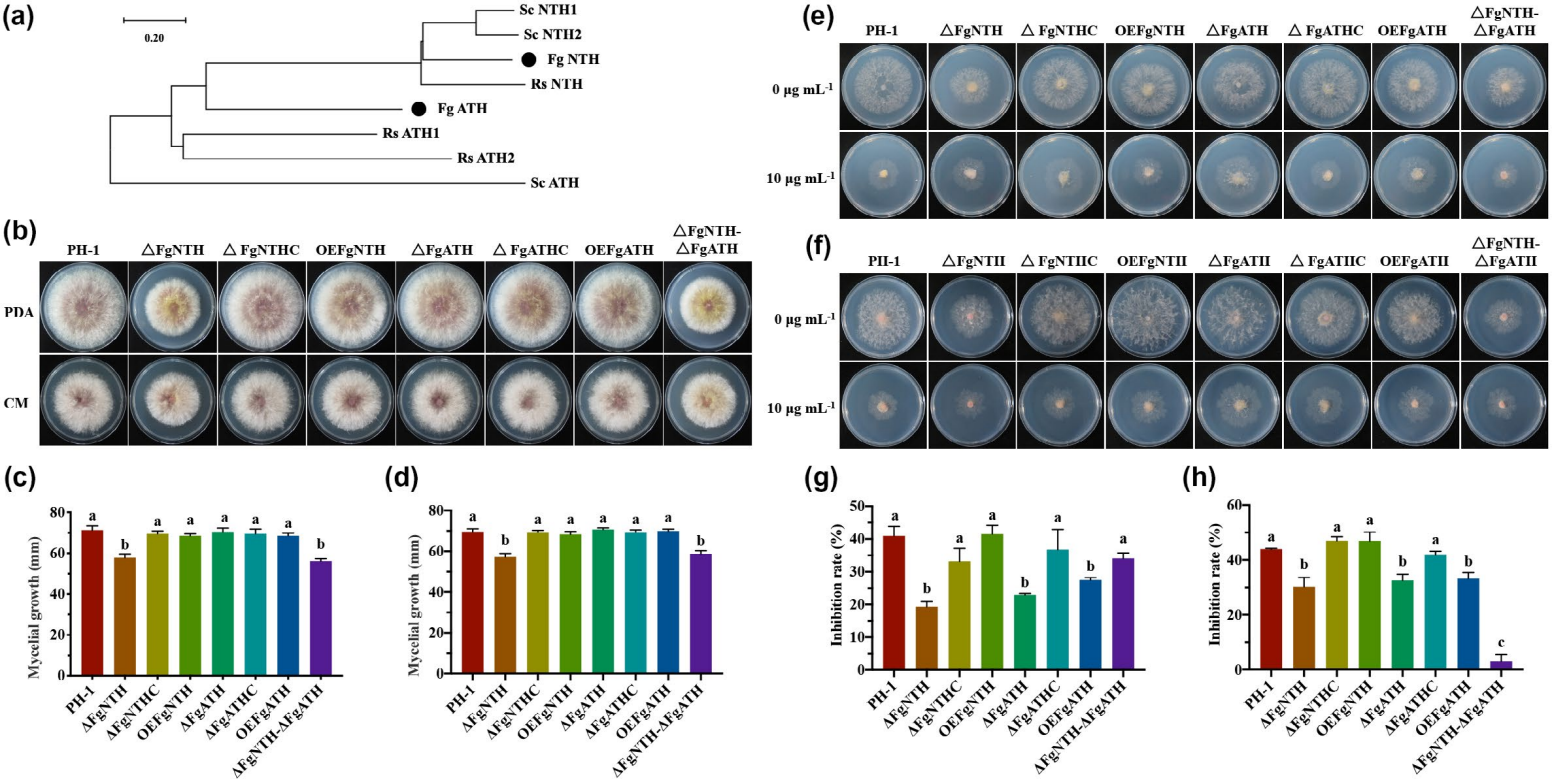
FgNTH and FgATH regulate the sensitivity to validamycin A (VMA) in *Fusarium graminearum*. (a) Phylogenetic analysis of NTH and ATH from *Rhizoctonia solani*, *Saccharomyces cerevisiae*, and *F. graminearum*. Abbreviations and accession numbers of the sequences are as follows: ScNTH1 (NP_010284.1), ScNTH2 (NP_009555.1), and ScATH (NP_015351.1) from *S. cerevisiae*; RsNTH (ELU40049.1), RsATH1 (ELU42236.1), and RsATH2 (ELU42026.1) from *R. solani*; FgNTH (FGSG_09895) and FgATH (FGSG_05622) from *F. graminearum*. The amino acid sequences of NTH and ATH were analysed using the neighbour‐joining method with the MEGA 8.0 program. (b, c, d) Colony morphology and mycelial growth of the wild‐type strain PH‐1, the single‐deletion mutants Δ*FgNTH* and Δ*FgATH*, the complemented strains of Δ*FgNTH*C and Δ*FgATH*C, the overexpression strains OE*FgNTH* and OE*FgATH*, and the double‐deletion mutant Δ*FgNTH*Δ*FgATH* on potato dextrose agar (PDA) and complete medium (CM) at 25 °C for 3 days. (e, f) Sensitivity of all strains to VMA on Czapek medium without carbon source and on Czapek medium containing trehalose as sole carbon source, respectively. Each strain was cultured on potato dextrose agar for 2 days and then transferred agar plugs (5 mm in diameter) containing mycelia from the colony margin onto the plates with VMA. Colony diameters were determined and pictures were taken after incubation for 4 days. (g) and (h) Inhibition rate of 10 μg/ml VMA to all strains on Czapek medium without carbon source and on Czapek medium containing trehalose as sole carbon source. Each test was independently performed three times. The data were analysed using by one‐way analysis of variance (ANOVA) and means were compared by the least significant difference at *p* < .05. The statistics and bar graphs were produced using GraphPad Prism v. 8.2

Using NTH and ATH of *Saccharomyces cerevisiae* as queries, we identified FgNTH (FGSG_09895) and FgATH (FGSG_05622) from the *F*. *graminearum* genome database using BLASTP. FgNTH and FgATH encoded 738‐ and 647‐amino acid proteins, exhibiting 54% and 42% identity to NTH and ATH of *S*. *cerevisiae*, respectively. Phylogenetic analysis for NTH and ATH from *F*. *graminearum*, *R*. *solani*, and *S*. *cerevisiae* was performed based on alignments of amino acid sequences and the results showed that FgNTH and NTH of *S*. *cerevisiae* were clustered into the same groups, while FgATH and ATH of *S*. *cerevisiae* were clustered into different groups (Figure 1a). Mutants of FgNTH and FgATH were conducted using a homology recombination strategy (Figures S1, S2, and S3).

### 2.2 FgNTH regulated vegetative growth in *F. graminearum*

**FIGURE 2:**
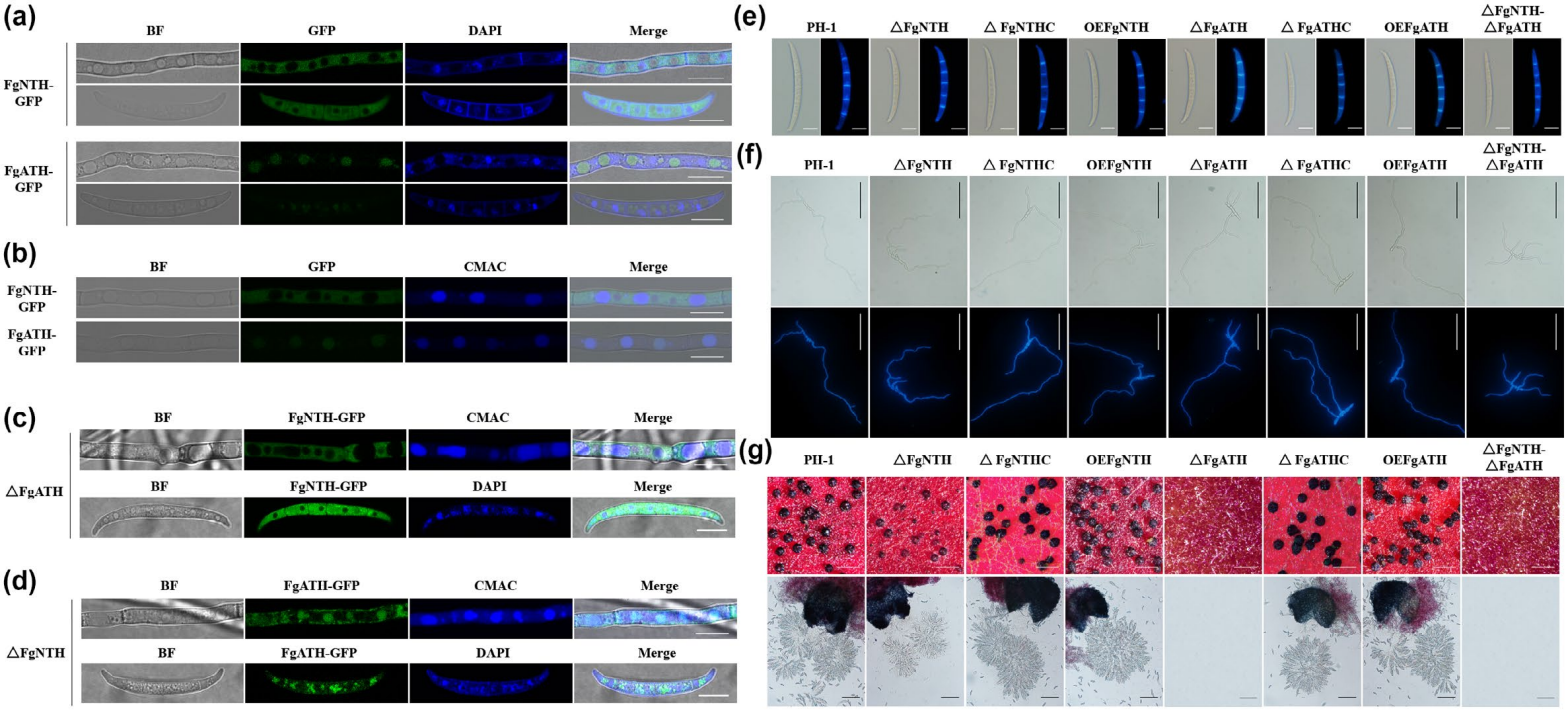
Roles of FgNTH and FgATH in modulating sexual reproduction and their subcellular localization in *Fusarium graminearum*. (a) The colocalization of FgNTH and FgATH with cell nucleus. (b) The colocalization of FgNTH and FgATH with vacuoles. The fusion strains FgNTH‐GFP and FgATH‐GFP were cultured in YEPD liquid medium for 1 day at 25 °C to harvest mycelia and in CMC medium for 3 days at 25 °C to produce conidia. The mycelia and conidia were stained with 4,6‐diamidino‐2‐phenylindole (DAPI) or 7‐amino‐4‐chloro‐methylcoumarin (CMAC) to observe fluorescence using a Leica TCS SP5 confocal microscope. GFP fusion protein: green fluorescence; DAPI and CAMC: blue fluorescence. Bar = 10 μm. (c) The localization of FgNTH in the mutant Δ*FgATH*. (d) The localization of FgATH in the mutant Δ*FgNTH*. The mycelia and conidia of strain Δ*FgATH FgNTH‐GFP* were harvested and stained with CMAC and DAPI, respectively, as described above. Bar = 10 μm. (e) Conidial morphology. Conidia were produced in CMC medium and stained with calcofluor white (CFW) to observe the septa using an Olympus IX‐71 inverted fluorescence microscope. Left image, bright field; right image, ultravioletlight excitation. Bar = 10 μm. (f) Conidial germination. Conidia were cultured in YEPD liquid medium for 8 hr and stained with CFW to observe the germination using an Olympus IX‐71 inverted fluorescence microscope. Top image, bright field; bottom image, ultraviolet light excitation. Bar = 20 μm. (g) Sexual reproduction. All strains were incubated on carrot medium for 14 days to produce perithecia. The perithecia and ascospores were observed using a Nikon SMZ25 fluorescence stereomicroscope and an Olympus IX‐71 inverted fluorescence microscope, respectively. Top image, perithecia, bar = 1 mm; bottom image, asci and ascospores, bar = 50 μm

The mycelial growth, conidiation, and conidial germination of the mutants were determined. The mutant Δ*FgNTH* grew significantly slower (*p* < .05) than the wild‐type strain PH‐1 on potato dextrose agar (PDA) and complete medium (CM), formed few aerial hyphae, and produced denser mycelia with shorter branching and few conidia, but no changes were observed with hyphal penetration, size, morphology, and septa number of conidia in comparison to the wild‐type strain PH‐1. The defects above were restored in the complemented strain Δ*FgNTH*C. Deletion mutant Δ*FgATH* and overexpression strains OE*FgATH* and OE*FgNTH* did not affect mycelial growth and conidiation. The double‐deletion mutant Δ*FgNTH*Δ*FgATH* showed similar biological phenotypes to the mutant Δ*FgNTH* (Figures 1a–d, 2e, S3c–f, and S5a–c). This indicated that *FgNTH*, not *FgATH*, plays a vital role in regulating vegetative growth in *F*. *graminearum*. Conidial germination was observed at 8 hr in the mutants and the wild‐type strain, and the number of germ tubes of conidia in the mutants Δ*FgNTH*, Δ*FgATH*, and Δ*FgNTH*Δ*FgATH* increased, but the germination rate decreased (Figures 2f and S3d). These results suggest that both *FgNTH* and *FgATH* are involved in conidial germination in *F*. *graminearum*.

### 2.3 FgNTH and FgATH regulated the sensitivity to validamycin A in *F. graminearum*

A previous study demonstrated that exogenous glucose antagonized the effects of VMA on *R*. *solani* growth (Robson et al., 1991). To clarify the targets of VMA at the gene level, the sensitivity of all mutants to VMA was tested on Czapek medium amended with different carbon sources. In Czapek medium without a carbon source, inhibition of growth by 10 µg/ml VMA was significantly lower (*p* < .05) for the strains Δ*FgNTH*, Δ*FgATH*, and OE*FgATH* than for the wild‐type strain PH‐1, but no difference was observed between Δ*FgNTH*Δ*FgATH* and the wild‐type strain PH‐1 (Figure 1e,g). In Czapek medium containing trehalose, inhibition of growth by 10 µg/ml VMA was significantly lower for the mutants Δ*FgNTH*, Δ*FgATH*, Δ*FgNTH*Δ*FgATH*, and strain OE*FgATH* than for the wild‐type strain PH‐1 (Figure 1f,h). In Czapek medium amended with glucose, inhibition of growth by 10 µg/ml VMA was significantly lower for the mutants Δ*FgNTH*, Δ*FgNTH*Δ*FgATH*, and strain OE*FgATH* than for the wild‐type strain PH‐1 (Figure S4a,c). In Czapek medium amended with starch, inhibition of growth by 10 µg/ml VMA was significantly lower for the mutants Δ*FgNTH*, Δ*FgATH*, and Δ*FgNTH*Δ*FgATH* than for the wild‐type strain PH‐1 (Figure S4b, d). These results indicated that both FgNTH and FgATH are targets of VMA, but their response to VMA is different under different carbon source conditions, and FgNTH appears to be the main target of VMA in *F*. *graminearum*.

### 2.4 Subcellular localization of FgNTH and FgATH

Yeast neutral and acid trehalases exhibit sharp differences in subcellular localization, being located in the cytosol and vacuoles or cell wall, respectively (Maicas et al., 2016). In this study, we fused *FgNTH* and *FgATH* with the green fluoresecent protein gene *GFP*, and transformed *FgNTH‐GFP* and *FgATH‐GFP* constructs into Δ*FgNTH* and Δ*FgATH*, respectively. As shown in Figure 2a,b, the FgNTH‐GFP and FgATH‐GFP fusion proteins were located into cytosol and vacuoles in hyphae and conidia, respectively. The distribution of FgATH‐GFP fusion protein onto cell wall was not observed in hyphae and conidia (Figure 2). As shown in Figure 2c, the FgNTH‐GFP fusion protein still located into the cytosol in hyphae and conidia in the mutant Δ*FgATH FgNTH*‐*GFP*, indicating that FgATH could not affect the distribution of FgNTH in *F*. *graminearum*. However, we observed that FgATH‐GFP fusion protein was located into the cytosol, vacuoles, and cell wall in hyphae and conidia of the mutant Δ*FgNTH FgATH‐GFP* (Figure 2d), strongly suggesting that the deletion of FgNTHaltered the distribution of FgATH in *F*. *graminearum*. Thus, we raised a hypothesis that FgNTH regulates the distribution of FgATH in *F*. *graminearum*.

### 2.5 FgNTH and FgATH are involved in sexual reproduction and virulence

The sexual reproduction of the mutants was observed on carrot agar plates. After incubating for 14 days, the mutant Δ*FgNTH* produced a few, immature perithecia and the mutant Δ*FgATH* failed to produce perithecia in comparison to the wild‐type strain PH‐1. The perithecial formation was restored in the complemented strains Δ*FgNTH*C and Δ*FgATH*C. There seemed to be no difference in perithecial production between the overexpression strains OE*FgNTH* and OE*FgATH* and the wild‐type strain PH‐1, as they all produced abundant and mature perithecia, asci, and ascospores (Figure 2g). In addition, no perithecia were seen with the double‐deletion mutant Δ*FgNTH*Δ*FgATH*, similar to the mutant Δ*FgATH* (Figure 2g). These results strongly indicate that FgNTH and FgATH are involved in sexual reproduction in *F*. *graminearum*, and FgATH is more essential than FgNTH.

**FIGURE 3:**
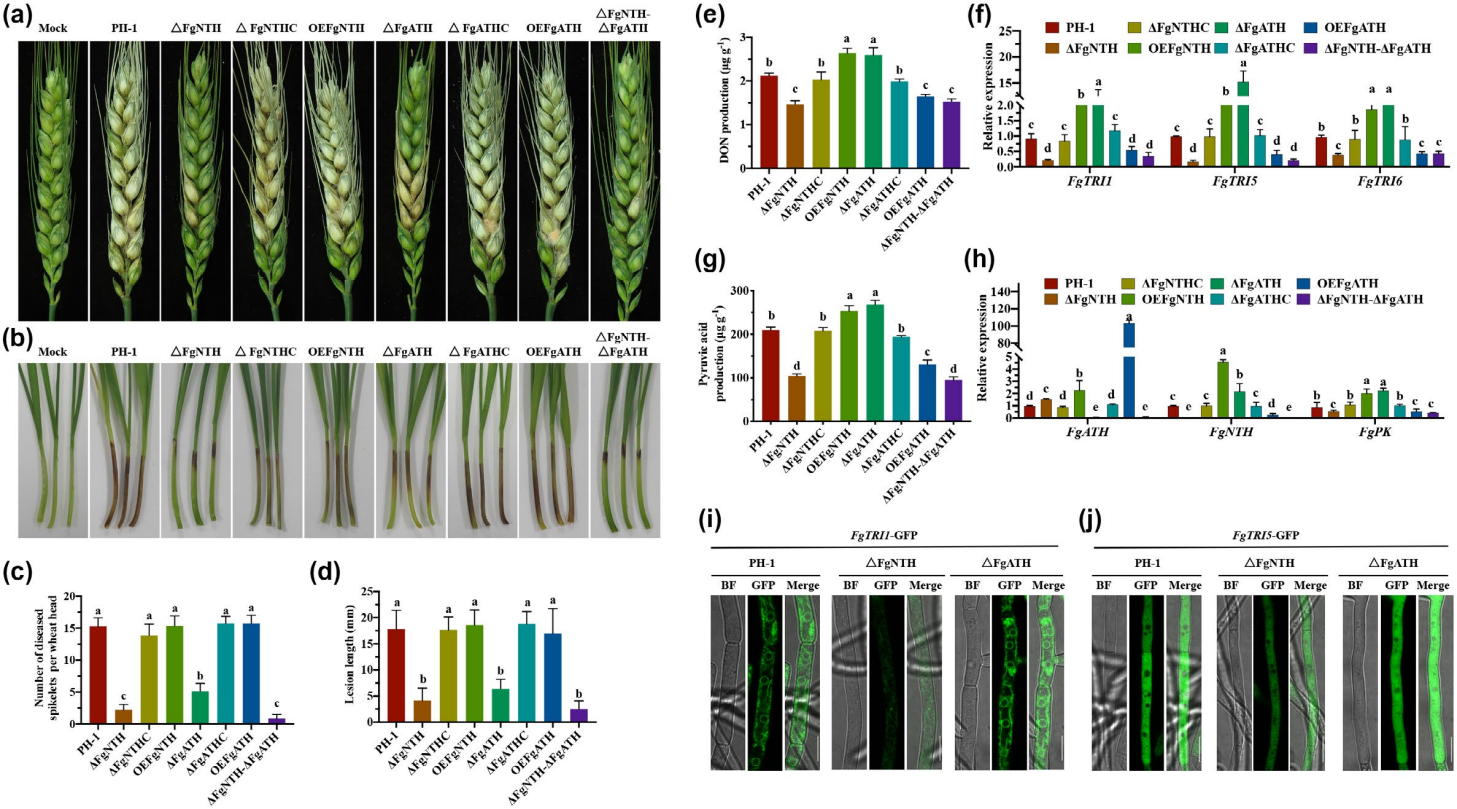
FgNTH and FgATH regulate virulence and deoxynivalenol (DON) biosynthesis in *Fusarium graminearum*. (a) Virulence on wheat heads 14 days after inoculation. The central spikelet of each wheat head was inoculated with 10 μl of conidial suspension. The controls were inoculated with sterile double‐distilled water. (b) Virulence on wheat coleoptiles 10 days after inoculation. The coleoptiles of 3‐day‐old wheat seedlings were cut and inoculated with 2 μl of conidial suspension. The controls were inoculated with sterile double‐distilled water. (c) Number of diseased spikelets. For the pathogenicity assay on wheat heads, each strain was inoculated to 15 wheat heads and the test was repeated twice. (d) Lesion length. For the pathogenicity assay on wheat coleoptiles, each strain was inoculated to 30 wheat coleoptiles and the test was repeated three times. (e) DON production. (f) The relative expression of *FgTRI1*, *FgTRI5*, and *FgTRI6*. (g) Pyruvate production. (h) The relative expression levels of *FgNTH*, *FgATH*, and *FgPK*. Fresh mycelia of each strain cultured in GYEP liquid medium for 3 days at 28 °C were harvested and used for determining the production of DON and pyruvate. These mycelia were also used for total RNA extraction and quantitative reverse transcription PCR assays. *FgActin* was used as the reference. (i) The fluorescence of fusion protein FgTRI1‐GFP. (j) The fluorescence of fusion protein FgTRI5‐GFP. The mycelia of fusion strains *FgTRI1‐GFP*, *FgTRI5‐GFP*, Δ*FgNTH FgTRI1‐GFP*, Δ*FgNTH FgTRI5‐GFP*, Δ*FgATH FgTRI1‐GFP*, and Δ*FgATH‐FgTRI5‐GFP* were cultured in GYEP liquid medium for 1 day at 28 °C and harvested for observing fluorescence using a Leica TCS SP5 confocal microscope. Bar = 10 μm. All assays were repeated three times independently. The data were analysed using one‐way analysis of variance (ANOVA) and means were compared by the least significant difference at *p* < .05. The statistics and bar graphs were produced using GraphPad Prism v. 8.2

We evaluated the virulence of the mutants in flowering wheat heads and wheat coleoptiles. At 14 days after inoculation on wheat heads, the mutants Δ*FgNTH* and Δ*FgATH* developed blight symptoms on fewer spikelets compared to the wild‐type strain PH‐1; the mutants OE*FgNTH* and OE*FgATH* developed typical scab symptoms affecting the inoculated spikelet to almost the entire head. The virulence was restored in the complemented strains Δ*FgNTH*C and Δ*FgATH*C (Figure 3a,c). The double deletion mutant Δ*FgNTH*Δ*FgATH* had similar virulence on wheat heads to the mutant Δ*FgNTH* (Figure 3a,c). Results of assays with wheat coleoptiles and cherry tomatoes were consistent with those with wheat heads (Figure 3b,d). These results revealed that both FgNTH and FgATH contribute to the virulence in *F*. *graminearum*.

### 2.6 Involvement of FgNTH in the response to stresses

As a central metabolic regulator, the trehalose pathway functions in stress responses in diverse organisms (Bonini et al., 2004). To investigate the stress sensitivity of FgNTH and FgATH, we examined the response of mutants of *FgNTH* and *FgATH* to NaCl, sorbitol, sodium dodecyl sulphate (SDS), H_2_O_2_, and Congo red. Compared with the wild‐type strain PH‐1, the mutants Δ*FgNTH* and Δ*FgNTH*Δ*FgATH* showed significantly decreased sensitivity to NaCl and sorbitol, and significantly increased sensitivity to SDS and H_2_O_2_, the strain OE*FgNTH* showed significantly decreased sensitivity to SDS and H_2_O_2_, and the mutant Δ*FgATH* and the strain OE*FgATH* showed similar sensitivity to NaCl, sorbitol, SDS, and H_2_O_2_ (Figure S6). Compared to the wild‐type strain PH‐1, the mutants Δ*FgNTH*, ΔF*gNTH*Δ*FgATH*, and OE*FgATH* had similar sensitivity to Congo red, while OE*FgNTH* and Δ*FgNTH*Δ*FgATH* had notably reduced sensitivity to Congo red (Figure S6). These results indicate that FgNTH contributes to resistance to osmotic, membrane, and oxidative stresses in *F*. *graminearum*.

### 2.7 FgNTH and FgATH regulate DON biosynthesis

We determined the trehalase activity and trehalose content in the mycelia of the mutants. FgNTH was essential for regulating the cytoplasmic trehalose and FgATH affected the hydrolysis of the cytoplasmic trehalose regulated by FgNTH (Figure S3g,h). To determine the effect of FgNTH and FgATH on regulating DON biosynthesis, we measured DON production of all the mutants in GYEP medium. Compared to wild‐type strain PH‐1, DON production was significantly decreased in Δ*FgNTH*, Δ*FgNTH*Δ*FgATH*, and OE*FgATH*, but significantly increased in Δ*FgATH* and OE*FgNTH*. No difference was observed between the complemented strains Δ*FgNTH*C and Δ*FgATH*C and the wild‐type strain PH‐1(Figure 3e). To further confirm DON biosynthesis, the expression levels of *FgTRI1*, *FgTRI5*, and *FgTRI6* genes were analysed by quantitative reverse transcription PCR (RT‐qPCR) in mycelia harvested from GYEP medium. The data showed that the relative expression levels of *FgTRI1*, *FgTRI5*, and *FgTRI6* were significantly decreased in Δ*FgNTH*, Δ*FgNTH*Δ*FgATH*, and OE*FgATH*, but were significantly increased in Δ*FgATH* and OE*FgNTH* (Figure 3f). The formation of *Fusarium* toxisomes containing DON biosynthesis enzymes, including TRI1, is vital to DON biosynthesis in *F*. *graminearum* (Tang et al., 2018). As shown in Figure 3i, the formation of *Fusarium* toxisomes was not affected in the mutants Δ*FgNTH* and Δ*FgATH*, but the fluorescence intensity of FgTRI1‐GFP was significantly reduced in the mutant Δ*FgNTH* and significantly increased in mutant Δ*FgATH* relative to the wild‐type strain PH‐1. In addition, the fluorescence intensity of FgTRI5‐GFP was also significantly reduced in the mutant Δ*FgNTH* and significantly increased in the mutant Δ*FgATH* relative to the wild‐type strain PH‐1 (Figure 3j). These results indicate that DON biosynthesis was positively regulated by FgNTH and negatively regulated by FgATH in *F*. *graminearum*.

Considering that pyruvate is the key molecule of the DON biosynthesis pathway, we analysed the production of pyruvate acid and the relative expression levels of the pyruvate kinase gene *FgPK* in GYEP medium. Compared to the wild‐type strain PH‐1, the production of pyruvate acid and the expression of *FgPK* were significantly reduced in the mutants Δ*FgNTH*, Δ*FgNTH*Δ*FgATH*, and OE*FgATH*, but were significantly increased in the mutants Δ*FgATH* and OE*FgNTH* (Figure 3g). In addition, we found that the expression level of *FgNTH* was significantly up‐regulated in mutant Δ*FgATH* and down‐regulated in OE*FgATH*; however, the expression level of *FgATH* was significantly up‐regulated in mutants Δ*FgNTH* and OE*FgNTH* (Figure 3h). These results further indicate that FgNTH positively, and FgATH negatively, regulated DON biosynthesis in *F*. *graminearum*, and the negative regulation effect of DON biosynthesis by FgATH depends on the expression level of FgNTH.

**FIGURE 4:**
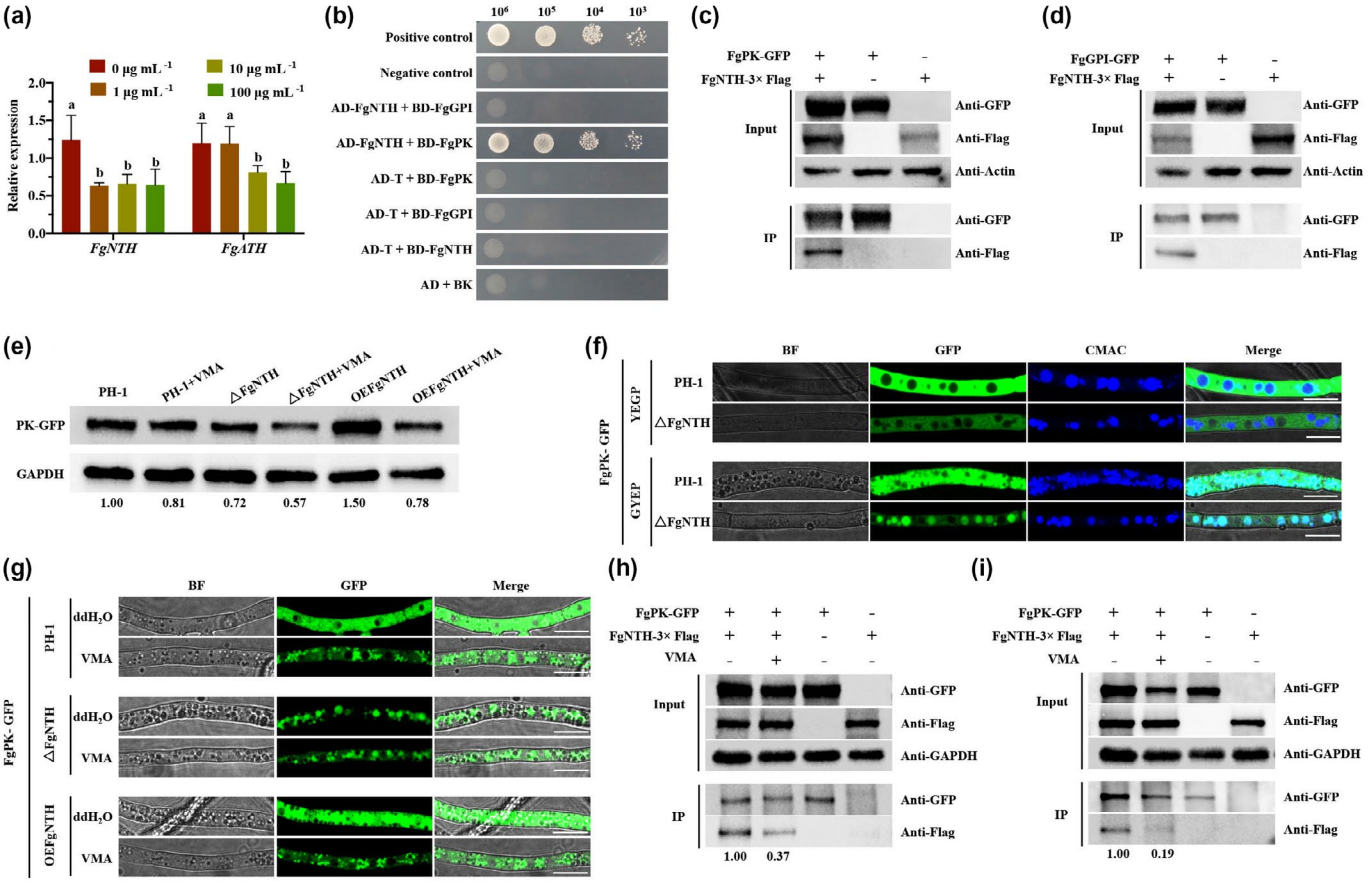
Validamycin A (VMA) reduces the interaction of FgNTH with FgPK in *Fusarium graminearum*. (a) VMA decreases the expression of *FgNTH* and *FgATH*. The wild‐type strain PH‐1 was cultured in GYEP liquid medium with 1, 10, and 100 μg/ml VMA at 28 °C for 3 days, and then used for quantitative reverse transcription PCR assays. (b) Yeast two‐hybrid analysis of the interaction between FgNTH and FgGPI, FgNTH, and FgPK. Different concentrations of the labelled yeast transformants were assayed for growth on SD−Trp−Leu−His plates. (c) Verification of FgNTH and FgPK interaction by coimmunoprecipitation (Co‐IP) assay. Total proteins (input) extracted from the strain containing FgNTH‐3 × FLAG FgPK‐GFP constructs or a single construct (FgNTH‐3 × FLAG or FgPK‐GFP) were subjected to sodium dodecyl sulphate polyacrylamide gel electrophoresis (SDS‐PAGE) and immunoblots were incubated with monoclonal anti‐GFP and monoclonal anti‐FLAG antibodies as indicated (upper image). In addition, each protein sample was pulled down using anti‐GFP antibodies with magnetic beads and further detected with monoclonal anti‐GFP and monoclonal anti‐FLAG antibodies (lower image). The protein samples were also detected with monoclonal anti‐actin antibody as a reference. (d) Verification of FgNTH and FgGPI interaction by Co‐IP assay. (e) VMA decreases the expression of FgPK. The fusion strains were cultured in GYEP liquid medium with or without 10 μg/ml VMA for 3 days at 28 °C, and were harvested for extracting the total proteins. Each lane was loaded with10 ng of total proteins for SDS‐PAGE, and further detected with monoclonal anti‐GFP antibody and monoclonal anti‐GAPDH antibody as a loading control. (f) FgNTH affects the localization of FgPK in GYEP medium. The fusion strains were cultured in YEPD or GYEP liquid medium for 3 days at 28 °C and then observed for fluorescence. Bar = 10 μm. (g) The location of FgPK‐GFP. The fusion strains were cultured in GYEP liquid medium with or without 10 μg/ml VMA for 3 days at 28 °C and were harvested to observe fluorescence. Bar = 10 μm. (h) VMA reduces the interaction between FgNTH and FgPK in YEPD medium. (i) VMA reduces the interaction between FgNTH and FgPK in GYEP medium. The fusion strain *FgNTH‐3 × FLAG FgPK‐GFP* was cultured in YEPD liquid medium with or without 10 μg/ml VMA for 1 day at 25 °C or in GYEP liquid medium with or without 10 μg/ml VMA for 3 days at 28 °C, and fresh mycelia were harvested for Co‐IP assay. All assays were repeated three times independently. The data were analysed using one‐way analysis of variance (ANOVA), and means were compared by the least significant difference at *p* < .05. The statistics and bar graphs were produced using GraphPad Prism v.8.2

To understand the response of FgNTH and FgATH to VMA under DON‐induced conditions, we determined the relative expression levels of *FgNTH* and *FgATH* in GYEP with or without VMA. Compared with the controls treated without VMA, the relative expression level of *FgNTH* was significantly down‐regulated by 1, 10, and 100 μg/ml VMA, and the relative expression of *FgATH* was significantly down‐regulated by 10 and 100 μg/ml VMA (Figure 4a). These results strongly demonstrate that both *FgNTH* and *FgATH* can respond to VMA, and *FgNTH* is more susceptible than *FgATH*. In other words, both *FgNTH* and *FgATH* are target genes of VMA but *FgNTH* is more essential than *FgATH* in terms of regulating DON biosynthesis.

### 2.8 FgNTH interacts with FgGPI and FgPK

Trehalases contribute to carbon metabolism and have other diverse regulatory effects on cell physiology (Barraza & Sanchez, 2013). In this study, we found that the growth deficiency of the mutant Δ*FgNTH* was not restored by glucose, fructose, or sucrose (Figure S7). We therefore speculated that FgNTH regulates the production of pyruvate and DON biosynthesis by interacting with enzymes in the glycolysis pathway to interfere with glycolysis. To assess this possibility, we conducted an affinity capture‐mass spectrometry assay to detect proteins that interact with FgNTH. The results showed that glucose‐6‐phosphate isomerase (GPI) and pyruvate kinase (PK) interacted with FgNTH. To confirm the results, we performed a yeast two‐hybrid (Y2H) assay, which clearly demonstrated an interaction between FgNTH and FgPK, but not between FgNTH and FgGPI (Figure 4b). We also subjected a strain bearing FgNTH‐3 × FLAG and FgPK‐GFP and a strain bearing FgNTH‐3 × FLAG and FgGPI‐GFP to a coimmunoprecipitation (Co‐IP) assay. The results of the assay confirmed the interaction between FgNTH and FgPK, and between FgNTH and FgGPI (Figure 4c,d). The results therefore indicated that FgNTH can directly interact with FgPK and can also indirectly interact with FgGPI in *F*. *graminearum*.

As shown in Figure S8, the fluorescence intensity of FgGPI‐GFP was not significantly different in Δ*FgNTH* and OE*FgNTH* with or without VMA. As shown in Figure 4g, the fluorescence intensity of FgPK‐GFP was decreased by VMA in the wild‐type strain PH‐1 and OE*FgNTH*, indicating that VMA reduced the expression of *FgPK* in *F*. *graminearum*. The results were also demonstrated by western blot (Figure 4e). In addition to the decrease in fluorescence intensity, we also found that FgPK‐GFP partially resides in the vacuole in Δ*FgNTH* under DON induction conditions (Figure 4f), implying that FgNTH affects the localization of FgPK‐GFP under DON induction conditions.

### 2.9 VMA reduces the interaction between FgNTH and FgPK

Given that a decrease of pyruvate production results in a reduction of DON biosynthesis, we examined whether VMA has an impact on the interaction of FgNTH and FgPK. The results showed that the interaction intensity between FgNTH and FgPK was significantly decreased when the strain *FgNTH‐3 × FLAG‐FgPK‐GFP* was cultured in YEPD medium with 10 µg/ml VMA (Figure 4h). Similarly, in DON induction medium (GYEP) containing 10 µg/ml VMA, the reduction in interaction intensity between FgNTH and FgPK was very significant (Figure 4i). These results indicate that VMA can reduce the interaction intensity between FgNTH and FgPK, interfering with the glycolysis pathway and reducing pyruvate production, thereby decreasing DON biosynthesis in *F*. *graminearum*.

### 2.10 FgNTH regulates the sensitivity to tebuconazole in *F. graminearum*

**FIGURE 5:**
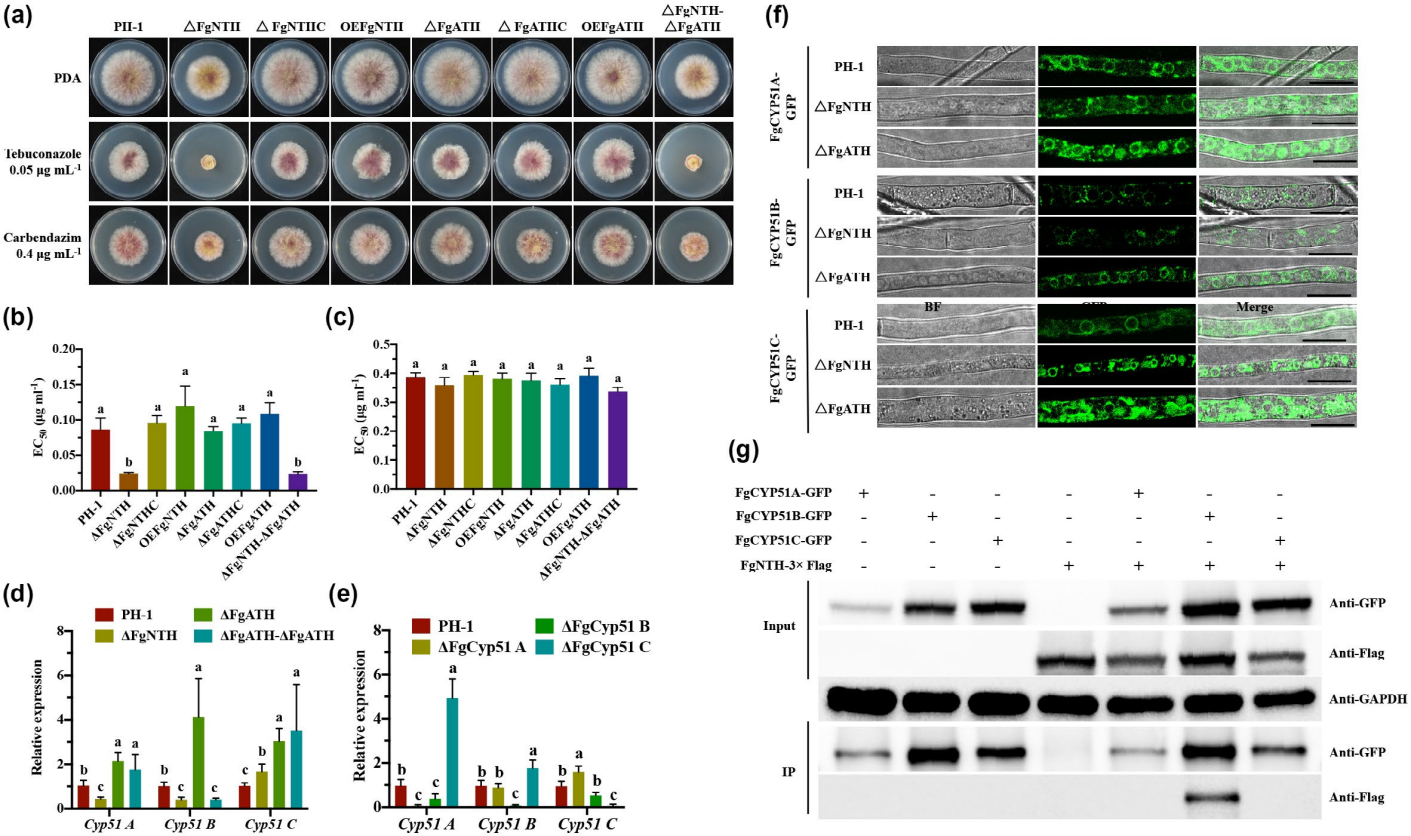
FgNTH regulates the sensitivity to tebuconazole in *Fusarium graminearum*. (a) Sensitivity of the mutant strains of *FgNTH* and *FgATH* to tebuconazole and carbendazim. The sensitivity of each strain to tebuconazole and carbendazim was determined on potato dextrose agar (PDA) based on mycelial growth inhibition and EC_50_ values. The concentration of tebuconazole was set to 0, 0.01, 0.05, 0.25, 0.625, and 1.25 μg/ml. The concentration of carbendazim was set to 0, 0.2, 0.4, 0.6, 0.8, and 1.0 μg/ml. (b) The EC_50_ values of each strain to tebuconazole. (c) The EC_50_ values of each strain to carbendazim. (d) *FgNTH* and *FgATH* affect the expression of *FgCYP51*. (e) *FgCYP51* expression in *FgCYP51* deletion mutants. Each strain was cultured in YEPD liquid medium for 2 days at 25 °C and was used for quantitative reverse transcription PCR assays. (f) The location of FgCYP51 in deletion mutants of *FgNTH* and *FgATH*. The fusion strains were cultured in YEPD liquid medium for 1 day at 25 °C and then the fluorescence was observed using a Leica TCS SP5 confocal microscope. Bar = 10 μm. (g) The interaction of FgNTH and FgCYP51B. Total proteins were eluted from anti‐GFP beads and detected with monoclonal anti‐GFP and monoclonal anti‐FLAG antibodies, respectively. Protein samples were detected with anti‐GAPDH antibody as a reference. Each test was independently determined three times. The data were analysed using one‐way analysis of variance (ANOVA) and means were compared by the least significant difference at *p* < .05. The statistics and bar graphs were produced using GraphPad Prism v. 8.2

We previously reported that, when applied with DMI fungicides, VMA has a synergistic effect in controlling FHB (Li et al., 2019). To explore the molecular mechanism of VMA synergizing with DMI fungicides, we assessed the sensitivities of the mutants to the currently extensively used fungicides tebuconazole and carbendazim. As indicated by the EC_50_ values, Δ*FgNTH* and Δ*FgNTH*Δ*FgATH* were more sensitive to tebuconazole than the wild‐type PH‐1 (Figure 5a–c). The results indicate that VMA has synergistic effects with tebuconazole.

Fungal sterol 14α‐demethylase enzymes (CYP51s) are the main target for DMI fungicides (Fan et al., 2013; Liu et al., 2011; Qian et al., 2018). In this study, the relative expression levels of *FgCYP51A*, *FgCYP51B*, and *FgCYP51C* were determined in Δ*FgNTH*, Δ*FgATH*, and Δ*FgNTH*Δ*FgATH*. Compared with the wild‐type strain PH‐1, *FgCYP51A* and *FgCYP51B* were significantly down‐regulated, but *FgCYP51C* significantly up‐regulated in Δ*FgNTH* (Figure 5d), *FgCYP51A*, *FgCYP51B*, and *FgCYP51C* were significantly up‐regulated in Δ*FgATH* (Figure 5d), and *FgCYP51A* and *FgCYP51C* were significantly up‐regulated, while *FgCYP51B* was still significantly down‐regulated in Δ*FgNTH*Δ*FgATH* (Figure 5d). This result indicated that FgNTH positively regulates *FgCYP51A* and *FgCYP51B*, which is the reason why VMA and DMIs have a synergistic effect. In addition, the expression levels of *FgCYP51A*, *FgCYP51B*, and *FgCYP51C* were also determined in Δ*FgCYP51A*, Δ*FgCYP51B*, and Δ*FgCYP51C*. Compared with PH‐1, *FgCYP51C* was up‐regulated and *FgCYP51B* was not changed in Δ*FgCYP51A*, *FgCYP51A* and *FgCYP51C* were down‐regulated in Δ*FgCYP51B*, and *FgCYP51A* and *FgCYP51B* were significantly up‐regulated in Δ*FgCYP51C* (Figure 5e). This result suggests that FgCYP51A negatively regulates *FgCYP51C*, FgCYP51B positively regulates *FgCYP51A* and *FgCYP51C*, and FgCYP51C negatively regulates *FgCYP51A* and *FgCYP51B*. Therefore, we presumed that FgNTH positively regulating FgCYP51A and FgCYP51B may be the result of the interaction of FgNTH and FgCYP51A or FgCYP51B.

To further explore the regulatory role of FgNTH and FgATH on FgCYP51A, FgCYP51B, and FgCYP51C, we constructed the fusion strains Δ*FgNTH FgCYP51A*‐*GFP*, Δ*FgNTH FgCYP51B‐GFP*, Δ*FgNTH FgCYP51C‐GFP*, Δ*FgATH FgCYP51A‐GFP*, Δ*FgATH FgCYP51B‐GFP*, and Δ*FgATH FgCYP51C‐GFP*. Fluorescence observation supports the result that FgNTH positively regulates *FgCYP51A* and *FgCYP51B* (Figure 5f). In addition, we analysed the result of FgNTH protein affinity capture‐mass spectrometry and found that FgNTH interacted with FgCYP51B. Furthermore, a Co‐IP assay was conducted to verify the interaction of FgNTH with FgCYP51B, indicating FgNTH interacted with FgCYP51B, not FgCYP51A or FgCYP51C. Based on the above results, we concluded that FgNTH positively regulates FgCYP51A and FgCYP51B by interacting with FgCYP51B, resulting in the synergistic effect of VMA and DMIs on *F*. *graminearum*.

## 3 DISCUSSION

*F*. *graminearum* causes some of the most economically important diseases of cereal crops and mycotoxin contamination in food and feed products, threatening our food supply and safety (Foroud & Eudes, 2009; Wegulo et al., 2015). Our laboratory recently found that VMA can be used to control FHB and to reduce DON contamination, and that its efficacy was improved when combined with azole fungicides (Li et al., 2019). However, the targets of VMA at gene level and its molecular mechanism of reducing DON contamination are poorly understood. Yeasts and filamentous fungi usually contain two types of trehalase that can be distinguished based on their cellular location, physiological role, and regulatory mechanism: the so‐called neutral trehalases and acid trehalases (Maicas et al., 2016). In the current study, we identified FgNTH and FgATH in *F*. *graminearum*, and found that FgNTH is well conserved, but FgATH has significant genetic distance among these fungi (Figure 1a).

In *Aspergillus nidulans* and *Neurospora crassa*, NTH catalyses intracellular trehalose breakdown and contributes some of the energy required for spore germination when external carbon is limited (Beltran et al., 2000; d’Enfert et al., 1999). In the entomopathogenic fungus *Beauveria bassiana*, NTH can respond to osmotic stress and thermal stress (Liu, Ying, et al., 2011). In the entomopathogenic fungus *Metarhizium acridum*, ATH contributes to in vivo growth and virulence (Jin et al., 2015). In *Candida parasilosis*, acid trehalase plays a major role in stress resistance and virulence (Sanchez‐Fresneda et al., 2014). In this study, we found that FgNTH and FgATH were required for conidial germination, sexual reproduction, and pathogenicity, and that FgNTH contributed to maintain vegetative growth and response to osmotic stress, membrane stress, and oxidative stress in *F*. *graminearum* (Figures 1, 2, 3, S3, S4, and S6). The functions of NTH and ATH in conidial germination and virulence of *F*. *graminearum* are consistent with their functions in other fungi, except that ATH in *F*. *graminearum* did not regulate the stress response as it does in *C*. *parasilosis*.

Yeast lacking the ability to produce ATH could not survive on the medium in which trehalose was the sole carbon source (Huang et al., 2007), but *F*. *graminearum* with a deletion of *FgATH* grew normally. In addition, NTH and ATH are essential for the sexual reproduction of *F*. *graminearum*. NTH has been found in the cytosol of many organisms, including *S*. *cerevisiae*, but ATH resides in the vacuole and is transported to the cell wall by the multivesicular body (Huang et al., 2007; Tran et al., 2016). In this study, we discovered that FgNTH was located in the cytosol and that FgATH was located in the vacuoles of hyphae and conidia (Figure 2a,b). Although the locations of FgNTH and FgATH in *F*. *graminearum* were generally consistent with their locations in other fungi, the fluorescence of FgATH‐GFP fusion protein was not observed in the cell wall. We also found that FgNTH affected the localization of FgATH, but that FgATH did not affect the localization of FgNTH (Figure 2). We therefore infer that NTH is more important than ATH in *F*. *graminearum*.

Potent inhibitors of trehalase might be useful as pesticides, and several trehalase inhibitors have been isolated from natural sources, including validamycin, validoxylamines, trehazolin, deoxynojirimycin, salbastain, and calystigin B4 (El Nemr & El Ashry el, 2011). Validamycin is a metabolite whose main component is VMA. In addition to controlling pathogenic fungi, VMA can enhance the resistance of plants (Li et al., 2019). Although VMA has been recognized as a potent trehalase inhibitor for many years, it was unclear before the current study whether NTH or ATH was its target. In this study, we identified both FgNTH and FgATH as the targets of VMA, but their responses to VMA were different under different carbon source conditions; FgNTH appears to be the main target of VMA in *F*. *graminearum* (Figures 1e–h and S5).

Pyruvate, a key intermediate in cellular metabolic pathways, is produced by glycolysis and is essential for DON biosynthesis in *F*. *graminearum* (Zhang et al., 2016). A previous study from our laboratory showed that the deletion of NTH reduced the production of pyruvate and DON in a wild‐type strain (isolate 2020) of *F*. *graminearum* (Li et al., 2019). In the current study, we also found that the FgNTH of wild‐type strain PH‐1 also positively regulated DON biosynthesis in *F*. *graminearum*. Moreover, we found that FgATH negatively regulated DON biosynthesis in *F*. *graminearum* (Figure 4e–h). *Fusarium* toxisomes play a critical role in DON biosynthesis, and FgTRI1 is localized in *Fusarium* toxisomes (Tang et al., 2018). FgTRI5 is the first committed step in the trichothecene biosynthetic pathway (Goswami & Kistler, 2004). In this study, we found that FgNTH and FgATH did not affect the formation of *Fusarium* toxisomes, but that FgNTH was a positive regulator of DON biosynthesis in *F*. *graminearum* and FgATH was a negative regulator owing to its effect on the expression of FgNTH (Figure 4).

Our previous laboratory study inferred that VMA reduces DON biosynthesis by inhibiting trehalase activity and thereby decreases the glucose content, leading to glucose shortage in *F*. *graminearum* (Li et al., 2019). However, abolishment of the trehalose synthesis pathway significantly down‐regulated the production of DON in *F*. *graminearum* (Song et al., 2014). In the current study, we found that the growth of the mutant Δ*FgNTH* was not promoted by glucose, fructose, or sucrose (Figure S7), which suggests that the deletion of *FgNTH* may restrict glycolysis rather than glucose shortage. In addition, the relative expressions of *FgNTH* and *FgATH* were significantly down‐regulated in the toxin‐inducing medium GYEP with VMA (Figure 5a). Based on the above results, we suspect that VMA reduces DON biosynthesis mainly by targeting FgNTH to interfere with glycolysis in *F*. *graminearum*.

Trehalases are involved in multiple regulatory processes: carbon partitioning and regulating bacterial viability in symbiotic interactions, and regulating chitin biosynthesis as well as the energy supply in the haemolymph for flight in insects (Barraza & Sanchez, 2013). However, these regulation roles were not confirmed at a molecular level. Trehalose is a well‐known stabilizing solvent of biological macromolecules and protects various enzymes from thermal inactivation (Das et al., 2019; Moruno Algara et al., 2019). PK plays a key role in glycolysis to control the metabolic flux (Schormann et al., 2019). Studies have shown that high concentration of trehalose inhibits the activity of PK by about 20% at 25 °C and protects pyruvate kinase from thermal inactivation at 60 °C (Guerrero‐Mendiola et al., 2009). In this study, we demonstrated that FgNTH directly interacted with FgPK and indirectly interacted with FgGPI in *F*. *graminearum* (Figure 5b–d). Moreover, FgNTH affected the localization of FgPK‐GFP under DON induction conditions (Figure 5f), and VMA decreased the expression of *FgPK* and reduced the interaction intensity between FgNTH and FgPK in *F*. *graminearum* (Figure 5e,h).

**FIGURE 6:**
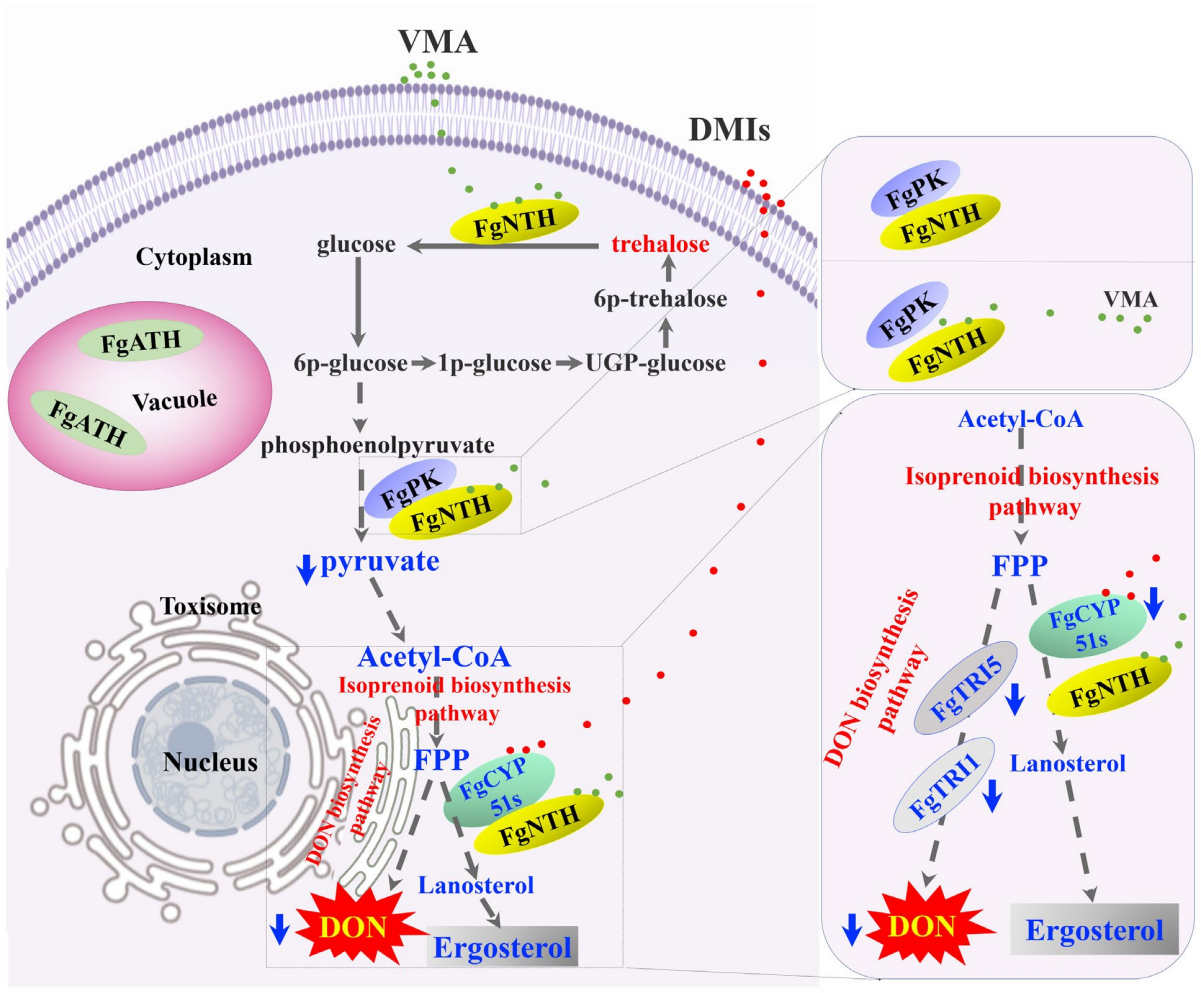
A schematic summary of validamycin A (VMA) inhibiting deoxynivalenol (DON) biosynthesis and synergizing with DMI fungicides against *Fusarium graminearum*. VMA inhibits FgNTH activity and reduces the expression level of *FgNTH*, which in turn reduces the interaction intensity between FgNTH and FgPK, interferingwith the glycolysis pathway to produce pyruvate and thus reducing DON biosynthesis. FgNTH can interact with FgCYP51B to reduce the expression level of *FgCYP51A* and *FgCYP51B*, resulting in the increasedsensitivity to DMIs in *F*. *graminearum*

The DMI fungicides, which are azole‐based compounds, block ergosterol biosynthesis and affect both membrane integrity and the function of membrane‐bound proteins in fungi (Mesterhazy et al., 2011; Qian et al., 2018; Strushkevich et al., 2010). However, owing to the broad use of various fungicides, resistance in pathogens has appeared and has become more serious. A tebuconazole‐resistant isolate of *F*. *graminearum* has been discovered in the United States (Spolti et al., 2014). Therefore, a reduction in the use of fungicides to delay the development of resistance is urgently needed. The biocontrol agents *Brevibacillus velezensis* and *Streptomyces* sp. are more tolerant to triazole fungicides, and it may be possible to combine these with triazole fungicides to control FHB (Palazzini et al., 2018). In this study, we found that trehalase‐deletion mutants were more sensitive to tebuconazole than the wild‐type PH‐1 (Figure 5a). This result is consistent with the previous finding that VMA has synergistic effects with DMI fungicides on controlling FHB in the field. *F*. *graminearum* has three *CYP51* genes. *FgCYP51B* encodes the enzyme primarily responsible for sterol 14α‐demethylation and is the most conserved *CYP51* gene (Fan et al., 2013; Qian et al., 2018). In this study, we found that FgNTH positively regulated the expression level of *FgCYP51A* and *FgCYP51B*, and it could interact with FgCYP51B. This result supports the finding that VMA has a synergistic effect with DMIs against *F*. *graminearum*. Taken together, we revealed the molecular mechanism of VMA inhibiting DON biosynthesis and synergizing with DMI fungicides against *F*. *graminearum*. Our data support the following model of how VMA reduces DON biosynthesis and has a synergistic effect with DMIs in *F*. *graminearum* (Figure 6): VMA inhibits FgNTH activity and reduces the expression level of *FgNTH*, which in turn reduces the interaction intensity between FgNTH and FgPK, interfering with the glycolysis pathway to produce pyruvate and thus decreasing DON biosynthesis; FgNTH can interact with FgCYP51B to reduce the expression level of *FgCYP51A* and *FgCYP51B*, resulting in the increased sensitivity to DMIs in *F*. *graminearum*.

## 4 EXPERIMENTAL PROCEDURES

### 4.1 Strains, culture conditions, and fungicides

*F*. *graminearum* wild‐type strain PH‐1 and all mutants in this study were stored at −4 °C and were routinely cultured at 25 °C on PDA. The strains used in this study are described in Table S1.

Validamycin A (98.5%) was purchased from Sangon Biotech Co. Ltd and was dissolved in distilled water. Carbendazim (98%) was provided by Jiangsu Lanfeng Biochemical Co. Ltd and was dissolved in 0.1 M HCl at 10 mg/ml as a stock solution. Tebuconazole (97%) was provided by Shandong Weifang Rainbow Chemical Co. Ltd and was dissolved in methanol at 10 mg/ml as a stock solution.

### 4.2 Generation of single‐deletion mutants Δ*FgNTH* and Δ*FgATH*, double‐deletion mutant Δ*FgNTH*Δ*FgATH*, and complemented strains Δ*FgNTH*C and Δ*FgATH*C

As shown in Figure S1, the single‐deletion mutants Δ*FgNTH* and Δ*FgATH* were generated by homologous replacement of the *FgNTH* and *FgATH* genes with double‐resistance genes, respectively (Liu et al., 2013). Flanking fragments about 1.2 kb upstream and downstream of the *FgNTH* and *FgATH* genes were amplified and then fused with the double‐resistance genes, conferring hygromycin phosphotransferase (*hph*) and herpes simplex virus‐thymidine kinase (*hsv‐tk*) fragments. The fusion fragments were transformed into the protoplasts of the wild‐type PH‐1 as previously described (Ge et al., 2013). The transformants were screened on PDA plates containing hygromycin B (100 μg/ml) and F2dU (0.2 μM), respectively. Putative Δ*FgNTH* and Δ*FgATH* mutants were analysed by PCR and Southern blot. Complemented strains Δ*FgNTH*C and Δ*FgATH*C were generated by homologous replacement of the double‐resistance gene fragments in Δ*FgNTH* and Δ*FgATH* with the full‐length fragments, including the upstream and downstream flanking fragments and target genes *FgNTH* and *FgATH*, respectively. The double‐deletion mutant Δ*FgNTH*Δ*FgATH* was generated by homologous replacement of the *FgATH* gene in the Δ*FgNTH* mutant with the G418 resistance gene *NEO*.

### 4.3 Generation of *FgNTH* and *FgATH* overexpression strains and GFP fusion strains

Overexpression strains OE*FgNTH* and OE*FgATH* were generated by complementing Δ*FgNTH* and Δ*FgATH*, respectively, with the fusion fragment carrying the *trpC* promoter (Figure S1). The construction of the fusion fragments was as follows: the upstream flanking region of *FgNTH* and *FgATH* genes was amplified and fused with the *trpC* promoter, and then further fused with the *FgNTH* gene and its downstream flanking fragment or *FgATH* gene and its downstream flanking fragment. The fusion fragments were transformed into Δ*FgNTH* and Δ*FgATH* mutants to replace the double‐resistance gene fragments. Putative overexpression strains OE*FgNTH* and OE*FgATH* were confirmed by gene sequencing, RT‐qPCR, and Southern blot.

We fused *FgNTH* and *FgATH* with *GFP* using the method described above, and transformed *FgNTH‐GFP* and *FgATH‐GFP* constructs into Δ*FgNTH* and Δ*FgATH* to generate *FgNTH‐GFP* and *FgATH‐GFP* fusion strains, respectively. *FgPK‐GFP*, *FgGPI‐GFP*, *FgTRI1‐GFP*, and *FgTRI5‐GFP* fusion strains were separately generated by transforming the fusion vector carrying the target genes into the wild‐type strain PH‐1. The construction of the fusion vector was as follows: the pYF11 vector was digested by the enzyme *Xho*I and was then cotransformed with target genes into yeast strains Sk1‐2S using the Alkali‐cation Yeast transformation kit (MP Biomedicals) to generate yeast strains carrying the fusion vector. Δ*FgNTH FgATH‐GFP* was generated by transforming the *FgATH‐GFP* fusion vector into Δ*FgNTH*, and Δ*FgATH FgNTH‐GFP* was generated by transforming the *FgNTH‐GFP* fusion vector into Δ*FgATH*. Fusion vectors *FgTRI1‐GFP* and *FgTRI5‐GFP* were transformed into Δ*FgNTH* and Δ*FgATH*, respectively, to generate strains Δ*FgNTH FgTRI1‐GFP*, Δ*FgNTH FgTRI5‐GFP*, Δ*FgATH FgTRI1‐GFP*, and Δ*FgATH FgTRI5‐GFP*. Fusion vectors *FgPK‐GFP* and *FgGPI‐GFP* were transformed into Δ*F* *gNTH* and OE*FgNTH*, respectively, to generate strains Δ*FgNTH FgPK‐GFP*, Δ*FgNTH FgGPI‐GFP*, OE*FgNTH FgPK‐GFP*, and OE*FgNTH FgGPI‐GFP*. These *GFP* fusion strains were confirmed by the presence of GFP signals.

### 4.4 Vegetative growth and conidiation assays

Colony morphology and mycelial growth were assessed on PDA and complete medium (CM), respectively (Kong et al., 2018). Hyphal tip branching was examined on cellophane with an Olympus IX‐71 inverted fluorescence microscope. Conidial production was measured by culturing four mycelial plugs of PDA in 20 ml of CMC medium for 4 days at 25 °C on a rotary shaker (175 rpm); the number of conidia was determined with a haemocytometer (Kong et al., 2018), the morphology and septa of conidia were examined by staining with calcofluor white (CFW) and observed with an Olympus IX‐71 inverted fluorescence microscope. The rate of conidial germination was assessed on water agar (agar 16 g/L) plates. The morphology of conidia germinating on YEP medium (tryptone 10 g/L, yeast extract 3 g/L, glucose 20 g/L) was observed with an Olympus IX‐71 inverted fluorescence microscope.

### 4.5 Sexual reproduction assay

For sexual development, strains were incubated on carrot medium (carrot 200 g/L, agar 16 g/L) for 3 days. Mycelia grown on carrot agar were scraped from the surface, 1 ml of 2.5% Tween 20 was added, and the mixture incubated under a near‐UV light (365 nm) at 25 °C (Shin et al., 2017). Ten days after sexual induction, the perithecia and ascospores were observed with a Nikon SMZ25 fluorescence stereomicroscope and an Olympus IX‐71 inverted fluorescence microscope, respectively.

### 4.6 Microscopic examinations of GFP fusion strains

Localization of FgNTH and FgATH was determined with strains *FgNTH‐GFP* and *FgATH‐GFP* grown on YEPD (yeast extract 3 g/L, peptone 10 g/L, glucose 20 g/L) (for hyphae) and CMC (for conidia); *FgNTH‐GFP* and *FgATH‐GFP* were examined with a Leica TCS SP5 confocal microscope. The hyphae and conidia of strains *FgNTH‐GFP* and *FgATH‐GFP* were stained with 4,6‐diamidino‐2‐phenylindole (DAPI) to observe nuclei. The hyphae of strains *FgNTH‐GFP*, *FgATH‐GFP*, Δ*FgNTH FgATH‐GFP*, Δ*FgATH FgNTH‐GFP*, and Δ*FgNTH FgPK‐GFP* were stained with 7‐amino‐4‐chloro‐methylcoumarin (CMAC) to observe vacuoles.

To detect the effect of FgNTH and FgATH on *Fusarium* toxisomes, the strains Δ*FgNTH FgTRI1‐GFP*, Δ*FgNTH FgTRI5‐GFP*, Δ*FgATH FgTRI1‐GFP*, and Δ*FgATH FgTRI5‐GFP* were cultured in GYEP medium for 3 days, and then the expression and location of FgTRI1 and FgTRI5 in their hyphae were assessed with a Leica TCS SP5 confocal microscope.

### 4.7 Pathogenesis and DON production assays

After the strains were cultured in CMC medium for 3 days, the conidia were collected by passing the medium through four layers of microscope lens paper. The filtrate was centrifuged at 3,381 × g for 5 min, and the pellet was resuspended in sterile distilled water to a final concentration of 10^6^ conidia/ml. The pathogenicity assay on the wheat heads was conducted by injecting 10 μl conidial suspension into a floret in the central section spikelet of a single flowering wheat head of the susceptible cultivar Huaimai 33 in the field (Kong et al., 2018). Wheat heads that were inoculated with sterile distilled water without conidia were used as controls and the wheat heads were assessed for disease after 14 days. The pathogenicity assay was also conducted on wheat coleoptiles by inoculating a cut wheat coleoptile with 2 μl conidial suspension (Li et al., 2019). Coleoptiles that were inoculated with sterile distilled water without conidia were used as controls and the coleoptiles were assessed for disease after 8 days at 25 °C.

To determine the quantity of DON produced by each strain, the strains were grown in liquid toxin‐inducing GYEP medium (yeast extract 1 g/L, peptone 1 g/L, sucrose 50 g/L). DON production was measured with the DON ELISA Plate Kit (Weisai) (Duan et al., 2020; Zhang et al., 2016). DON production in vitro was expressed as a ratio of DON content to dry mycelial weight (μg/g). The experiment was performed three independent times.

### 4.8 Fungicides and osmotic sensitivity assays

The sensitivity of all mutants to VMA was measured on Czapek medium without sugar (NaNO_3_ 3 g/L, K_2_HPO_4_ 1 g/L, KCl 0.5 g/L, MgSO_4_.7H_2_O 0.5 g/L, FeSO_4_.7H_2_O 0.01 g/L, agar 16 g/L) or on Czapek medium with 2% glucose, 2% trehalose, and 1% starch. The sensitivity of all mutants to carbendazim and tebuconazole was determined on PDA based on mycelial growth inhibition and EC_50_ values (Li et al., 2019). To determine the response of strains to stresses, the strains were grown on PDA alone or PDA containing final concentrations of 1.2 M NaCl, 1.2 M sorbitol, 0.05% H_2_O_2_, 0.025% SDS, or 0.05% Congo red (Chen et al., 2018).

### 4.9 Trehalase activity, trehalose, and pyruvate production

For each strain, three agar blocks containing mycelia (5 mm in diameter) were cultured in 25 ml YEPD medium with rotation (175 rpm), and the fresh mycelia were harvested after 3 days at 25 °C. Trehalase activity was measured with the Trehalase Assay Kit (Solarbio) according to the manufacturer's instructions, as was trehalose production (Li et al., 2019). For pyruvate production, three agar blocks containing mycelia (5 mm in diameter) were cultured in 20 ml GYEP with rotation (175 rpm). Trehalose production content was expressed as a ratio of trehalose production to fresh mycelial weight (mg/g). After 3 days at 28 °C, the fresh mycelia were harvested and pyruvate production was detected with the Pyruvate Assay Kit (Solarbio) according to the manufacturer's instructions (Li et al., 2019). Pyruvate production content was expressed as a ratio of pyruvate production to fresh mycelial weight (μg/g).

### 4.10 DNA extraction and PCR

Genomic DNA was extracted from mycelia using the cetyltrimethylammonium bromide (CTAB) method (Leslie & Summerell, 2006). The primers used in this study were synthesized by an oligonucleotide synthesis facility (GenScript Biotechnology Co. Ltd). General PCR was performed following the instructions of DNA polymerase (Vazyme Biotech Co. Ltd). All primers used for PCR are listed in Table S2.

### 4.11 RNA extraction and RT‐qPCR assays

To determine the relative expression level of genes associated with DON biosynthesis, mycelia of all strains were cultured in GYEP liquid medium for 4 days at 28 °C in the dark. Total RNA was extracted using the TIANGEN RNA simple Total RNA Kit (Tiangen Biotech) and was reverse transcribed to cDNA using the HiScript II Q RT SuperMix for qPCR (+gDNA wiper) (Vazyme Biotech Co. Ltd). qPCR was performed with a CFX96 Real‐Time System (Bio‐Rad Laboratories). The expression of the genes *FgTRI1*, *FgTRI5*, *FgTRI6*, and *FgPK* was determined, and the *FgActin* gene was used as a reference. The experiment was performed three independent times.

### 4.12 Affinity capture‐mass spectrometry analysis

Expression of GFP‐tagged FgNTH in strain *FgNTH‐GFP* was confirmed by western blot analysis using anti‐GFP antibody. Strain *FgNTH‐GFP* was cultured in YEPD liquid medium with shaking (175 rpm). After 36 hr at 25 °C, mycelia were harvested and total proteins were extracted using RIPA extraction buffer (50 mM Tris‐HCl [pH 7.5], 150 mM NaCl, 5 mM EDTA, 1% Triton X‐100, 1 mM protease inhibitor cocktail [Yeasen Biotech Co. Ltd], and 1 mM phenylmethylsulfonyl fluoride [PMSF]). After protein extraction, the supernatant was incubated with 25 μl GFP‐Trap_MA beads (ChromoTek) at 25 °C for 1 hr, and was then washed three times with wash buffer (10 mM Tris‐HCl [pH 7.5], 150 mM NaCl, and 0.5 mM EDTA). The proteins were eluted with 50 μl SDT (4% SDS, 100 mM Tris‐HCl [pH 7.6], and 100 mM dithiothreitol) and were then subjected to mass spectrometry analysis (Shanghai Applied Protein Technology Co. Ltd). The putative FgNTH‐interacting proteins are listed in Table S3.

### 4.13 Yeast two‐hybrid assays

Yeast two‐hybrid assays (Y2H) were conducted as previously described (Ren et al., 2018). The coding sequence of each tested gene was amplified from the cDNA of the wild‐type strain PH‐1 and inserted into the yeast GAL4‐binding domain vector pGBKT7 and the GAL4‐activation domain vector pGADT7 (Clontech). The pairs of plasmids were cotransformed into the yeast reporter strain AH109 following the LiAc/SS‐DNA/PEG transformation protocol (Schiestl & Gietz, 1989). Plasmid pairs pGBKT7‐53 and pGADT7 served as a positive control, and the plasmid pairs pGBKT7‐Lam and pGADT7 served as a negative control. The yeast transformants were grown at 30 °C for 3 days on synthetic dropout medium (SD) lacking Leu and Trp, and were then transferred to SD without His, Leu, or Trp and containing 5 mM 3‐aminotriazole (3‐AT) to check binding activity. This experiment was performed three independent times.

### 4.14 Western blot assays and Co‐IP assays

Total protein of fresh mycelia (100 mg) was extracted with 1 ml RIPA extraction buffer and 10 μl protease inhibitor cocktail (Gu et al., 2015). The protein sample was mixed with loading buffer and boiled for 10 min before 10 μl of the sample was separated by 10% SDS polyacrylamide gel electrophoresis (SDS‐PAGE) and transferred onto a polyvinylidene fluoride membrane with a Bio‐Rad electroblotting apparatus.

The *FgNTH‐3 × FLAG* fusion strain was generated by complementing the Δ*FgNTH* deletion mutants with a fusion fragment carrying the *3 × FLAG* gene. *FgGPI‐GFP* and *FgPK‐GFP* fusion constructs were transformed into the *FgNTH‐3 × FLAG* fusion strain to generate double‐label strains *FgNTH‐3 × FLAG‐FgPK‐GFP* and *FgNTH‐3 × FLAG‐FgGPI‐GFP*. All strains carrying label were confirmed by western blot analyses. For Co‐IP assays, total proteins were extracted and incubated with GFP‐Trap_MA beads. Proteins eluted from the agarose were analysed by western blotting with monoclonal anti‐FLAG 390002 (Zenbio) and monoclonal anti‐GFP antibodies 300943 (Zenbio), respectively. Each protein sample was also detected with anti‐actin antibody 700068 (Zenbio) or anti‐GAPDH antibody 60004–1‐Ig (Proteintech) as a reference. Incubation with a secondary antibody and chemiluminescent detection were performed as described previously (Zhou et al., 2020).

### 4.15 Statistical analysis

Each treatment was represented by three independent repetitions. Statistical analysis was performed using one‐way of variance (ANOVA), followed by the Tukey’s multiple comparison test. The level of significance was set at α = .05. The statistics and bar graphs were produced using a GraphPad Prism v. 8.2 (Graph Pad Software).

## 5 COMPETING INTERESTS

The authors declare no competing interest.

## Data Availability

The data that support the findings of this study are available from the corresponding author upon reasonable request.
