# Supplementary material for: Mechanism of validamycin A inhibiting DON biosynthesis and synergizing with DMI fungicides against Fusarium graminearum
Source: Mol Plant Pathol. 2021 May 2;22(7):769–85. doi: 10.1111/mpp.13060 (PMC8232029; doi:10.1111/mpp.13060)
Supplement: Supplementary file 1 [file MPP-22-769-s004.docx]

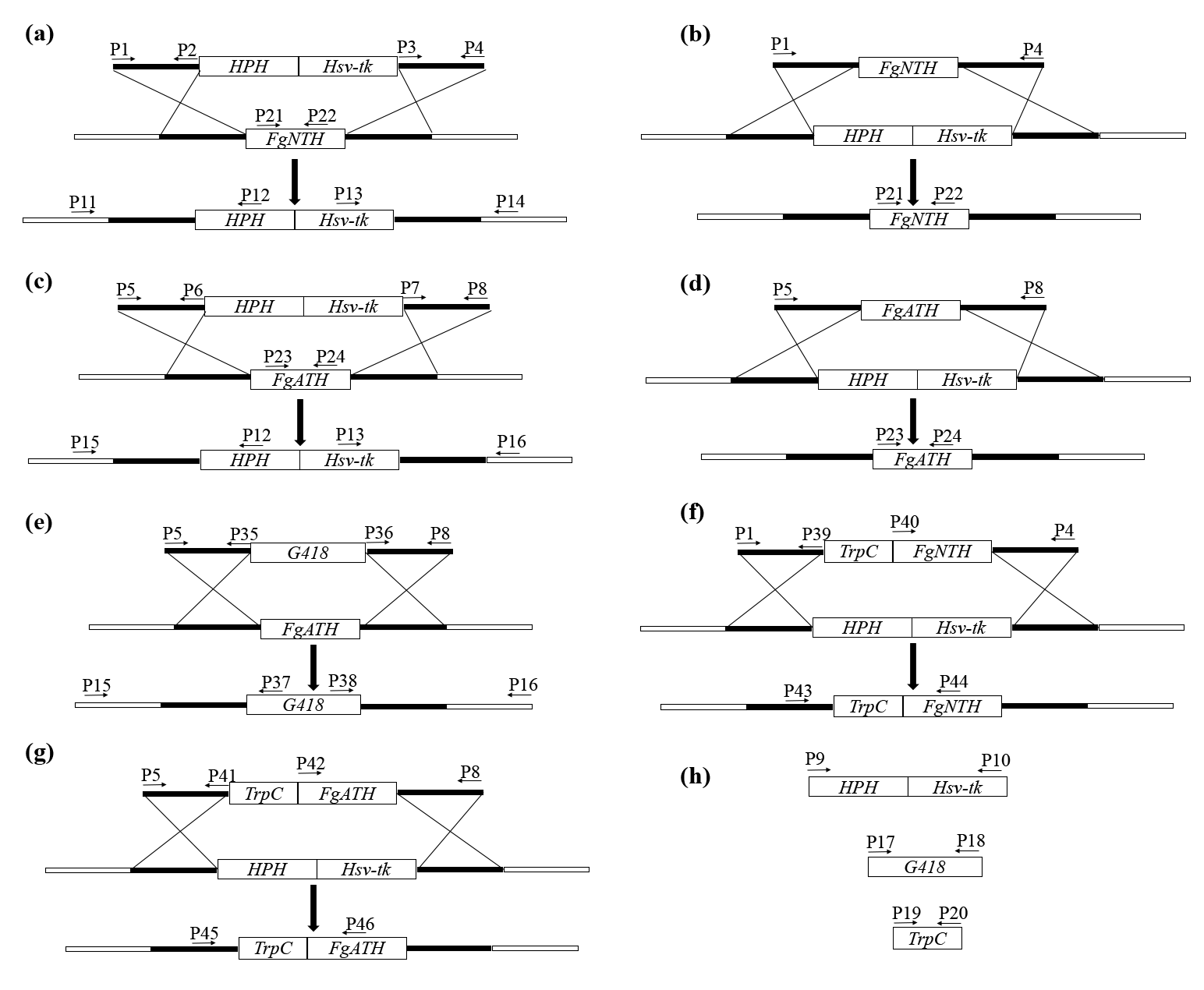


**Fig. S1 Generation of the mutant strains of FgNTH and FgATH. (a)** The deletion of FgNTH*.* The △FgNTH were constructed via a homologous replacement strategy. Primer pairs P1/P2 and P3/P4 were used to amplify the flanking fragments about 1.2kb upstream and downstream of *FgNTH*. Primer pairs P21/P22 was used to specifically amplify the partial fragment of *FgNTH* (746 bp). Primer pairs P11/P12 and P13/P14 were used to verified the upstream (2248 bp) and downstream (2300 bp) binding fragments, respectively. **(b)** Generation of the complement strain △FgNTHC*.* Primer pairs P1/P4 was used to amplify a fragment including the *FgNTH* gene with the flanking fragments of *FgNTH*. **(c)** The deletion of FgATH. The △FgATH were constructed via a homologous replacement strategy. Primer pairs P5/P6 and P7/P8 were used to amplify the flanking fragments about 1.2kb upstream and downstream of FgNTH. Primer pairs P23/P24 was used to specifically amplify the partial fragment of *FgATH* (718 bp). Primer pairs P15/P12 and P13/P16 were used to verified the upstream (2279 bp) and downstream (2385 bp) binding fragments, respectively. **(d)** Generation of the complement strain △FgATHC*.* Generation of the complement strain △FgATHC*.* Primer pairs P5/P8 was used to amplify a fragment including the *FgATH* gene with the flanking fragments of *FgATH*. **(e)** The double-gene deletion of FgNTH and FgATH. In the mutant △FgNTH, FgATH was replaced by resistance gen G418 via a homologous replacement strategy. Primer pairs P5/P35 and P36/P8 were used to amplify the flanking fragments about 1.2kb upstream and downstream of *FgATH*. **(f)** The overexpression of FgNTH*.* Overexpression strain OEFgNTH was generated by complementing △FgNTH with the fusion fragment carrying the trpC promoter. **(g)** The overexpression of FgATH*.* Overexpression strain OEFgATH was generated by complementing △FgATH with the fusion fragment carrying the trpC promoter. **(h)** The amplification of resistance gene *HPH-Hsv-tk*, *G418*, and *TrpC* promoter fragment. *HPH-Hsv-tk*: hygromycin phosphotransferase (hph) and herpes simplex virus-thymidine kinase (hsv-tk) fragments, *G418:*geneticin resistance gene.
