# Supplementary material for: Mechanism of validamycin A inhibiting DON biosynthesis and synergizing with DMI fungicides against Fusarium graminearum
Source: Mol Plant Pathol. 2021 May 2;22(7):769–85. doi: 10.1111/mpp.13060 (PMC8232029; doi:10.1111/mpp.13060)
Supplement: Supplementary file 2 [file MPP-22-769-s014.docx]

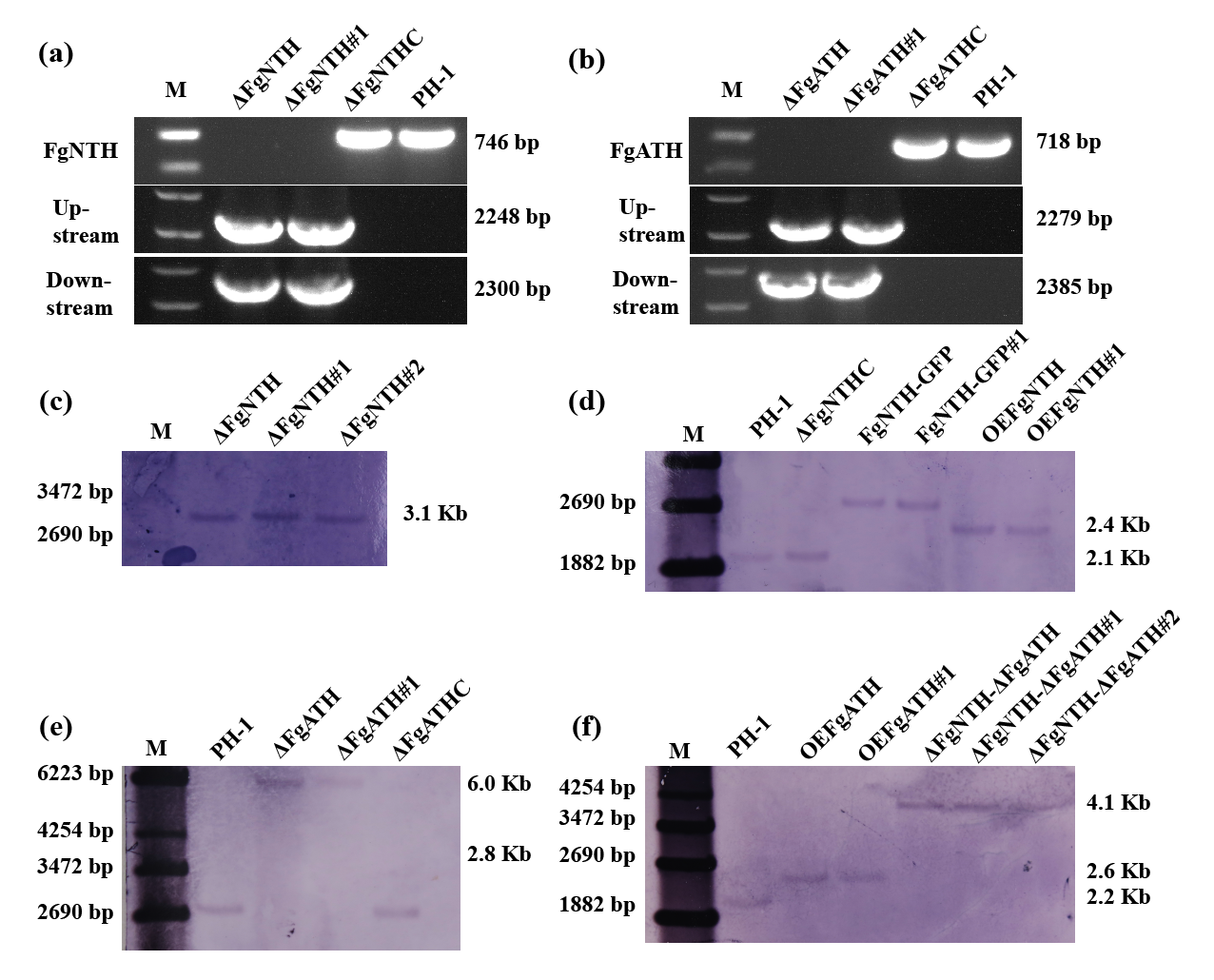


**Fig. S2** **The verification of the mutant strains of FgNTH and FgATH.** **(a)** The deletion and complement of FgNTH were verified by PCR. △FgNTH and △FgNTH#1 were the deletion mutants of FgNTH, △FgNTHC was the complemented transformant of △FgNTH. **(b)** The deletion and complement of FgATH were verified by PCR. △FgATH and △FgATH#1 were the deletion mutants of FgATH, △FgATHC was the complemented transformant of △FgATH. **(c and d)** The deletion mutants, complement, overexpression, and GFP fusion strains of FgNTH were further verified by southern blot. Total DNA of all mutants and the wild type strain PH-1 were extracted using cetyltrimethylammonium bromide (CTAB), and then were digested by restriction endonuclease *EcoR* V. Southern blot was analyzed with the probe located in up-stream of FgNTH amplificated by the primers P31/P32. **(e)** The deletion and complement transformants of FgATH were further verified by southern blot. Total DNA of the transformants was extracted as described above and were digested by restriction endonuclease *Hind* III. Probe was located in down-stream of FgATH amplificated by the primers P47/P48. **(f)** The overexpression strains of FgATH and the double deletion mutants of FgNTH and FgATH were further verified by southern blot. Total DNA was also digested by *Hind* III. Probe was located in up-stream of FgATH amplificated by the primers P33/P34.
