# Supplementary material for: Mechanism of validamycin A inhibiting DON biosynthesis and synergizing with DMI fungicides against Fusarium graminearum
Source: Mol Plant Pathol. 2021 May 2;22(7):769–85. doi: 10.1111/mpp.13060 (PMC8232029; doi:10.1111/mpp.13060)
Supplement: Supplementary file 3 [file MPP-22-769-s013.docx]

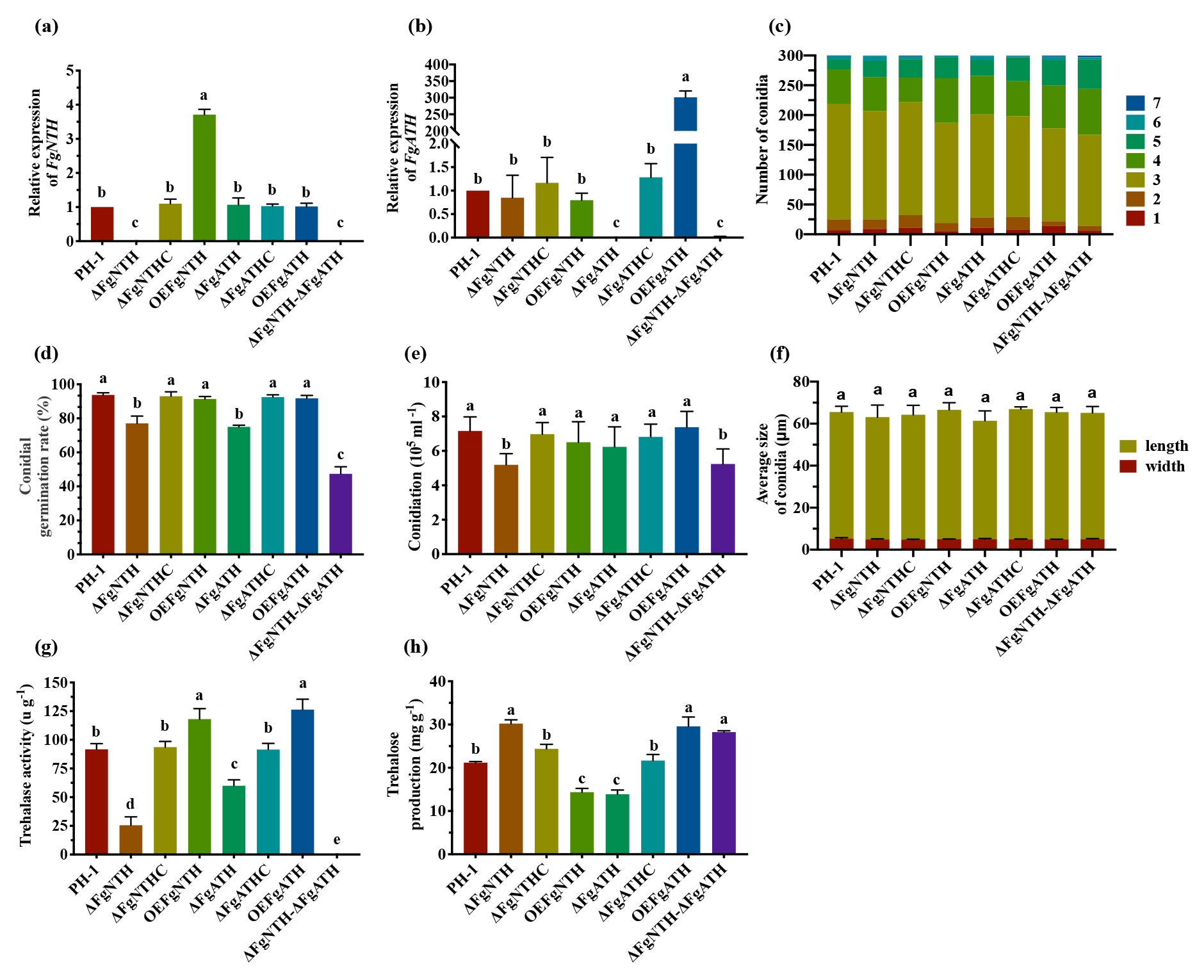


**Fig. S3 Requirements of FgNTH and FgATH for conidiation, conidial germination, and hydrolysis of trehalose.** **(a, b)** The relative expression of *FgNTH* and *FgATH* in the mutant strains of FgNTH and FgATH on YEPD liquid medium at 25ºC for 2 days (175 rpm). **(c)** Septa number of conidia. **(d)** Conidial germination rate. **(e)** Conidiation. **(f)** Average size of conidia.

Conidia of each strain produced in CMC medium at 25ºC for 5 days, and then conidiation were counted with hemocytometer. Conidia size were determine using Image J software by an Olympus IX-71 inverted fluorescence microscope (Tokyo, Japan), three hundred conidia were counted for calculate average size. Conidia germination rate was conducted on water agar plate. **(g)** trehalase activity. **(h)** trehalose production. Fresh mycelia cultured in YEPD liquid medium at 25ºC for 3 days were harvested for determining the trehalase activity and trehalose production. Each test was independently determined three times. The data were statistically analyzed using by one-way analyses of variance (ANOVA), and means were compared by the least significant difference at P < 0.05. The statistics and bar graphs were performed using GraphPad Prism 8.2.
