# Supplementary material for: Mechanism of validamycin A inhibiting DON biosynthesis and synergizing with DMI fungicides against Fusarium graminearum
Source: Mol Plant Pathol. 2021 May 2;22(7):769–85. doi: 10.1111/mpp.13060 (PMC8232029; doi:10.1111/mpp.13060)
Supplement: Supplementary file 4 [file MPP-22-769-s015.docx]

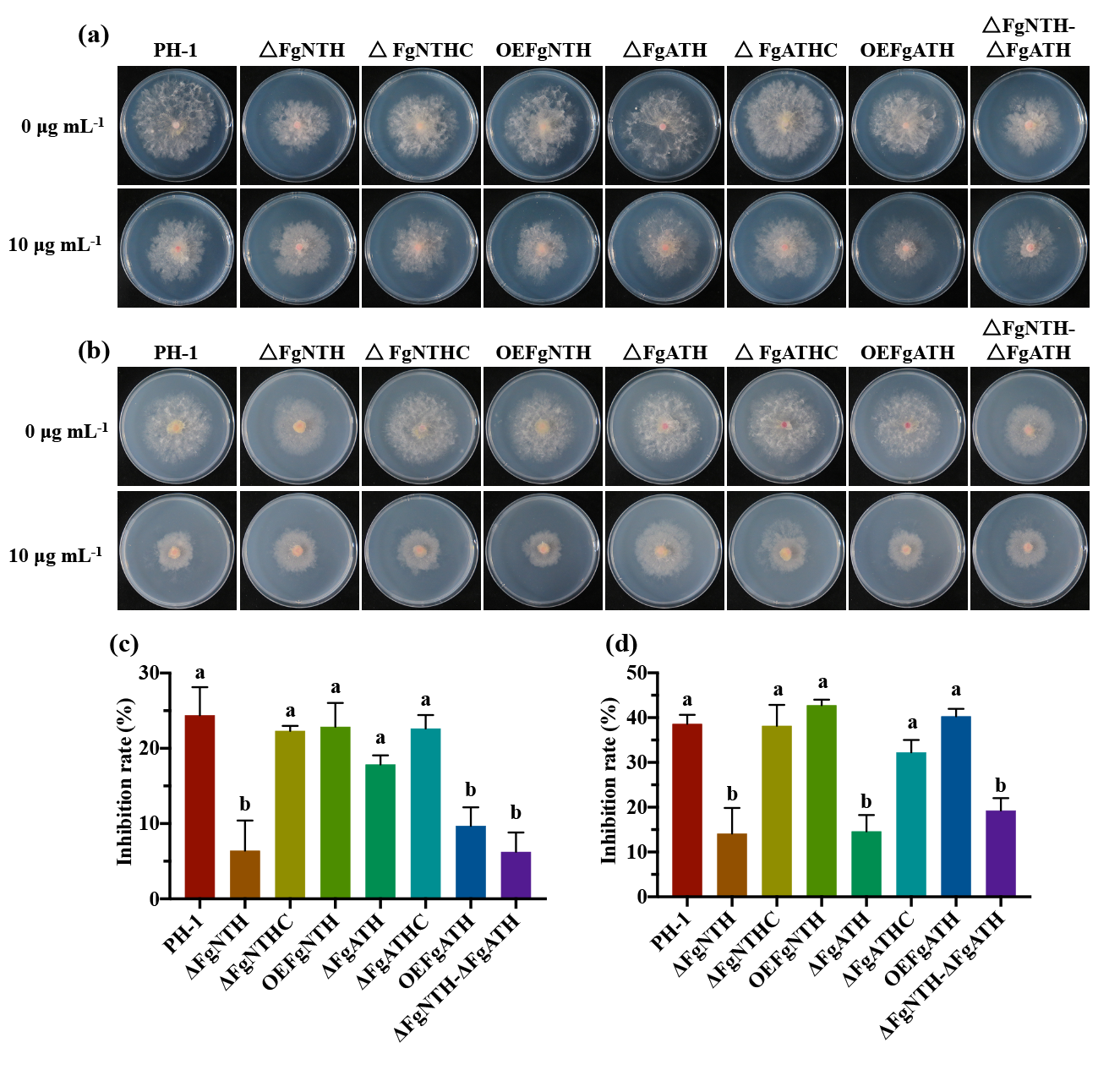


**Fig. S4 Sensitivity of the mutant strains of FgNTH and FgATH to VMA on Czapek medium with glucose or starch.**

**(a)** Sensitivity of all strains to VMA on Czapek medium with glucose. Each strain was cultured on PDA medium for 2 days, and then transferred agar plugs (5 mm in diameter) containing mycelia of colony margin to Czapek medium with glucose containing 0, 1, 10 and 100 μg mL^-1^ VMA. Colony diameters were determined and pictures were taken after inoculated 4 days.

**(b)** Sensitivity of all strains to VMA on Czapek medium with starch. Each strain was cultured on PDA medium for 2 days, and then transferred agar plugs (5 mm in diameter) containing mycelia of colony margin to Czapek medium with starch containing 0, 1, 10 and 100 μg mL^-1^ VMA. Colony diameters were determined and pictures were taken after inoculated 5 days.

**(c)** Inhibition rate of 10 μg mL^-1^ VMA to all strains on Czapek medium with glucose. **(d)** Inhibition rate of 10 μg mL^-1^ VMA to all strains on Czapek medium with starch. Each test was independently determined three times. The data were statistically analyzed using by one-way analyses of variance (ANOVA), and means were compared by the least significant difference at P < 0.05. The statistics and bar graphs were performed using GraphPad Prism 8.2.
