# Supplementary material for: Mechanism of validamycin A inhibiting DON biosynthesis and synergizing with DMI fungicides against Fusarium graminearum
Source: Mol Plant Pathol. 2021 May 2;22(7):769–85. doi: 10.1111/mpp.13060 (PMC8232029; doi:10.1111/mpp.13060)
Supplement: Supplementary file 5 [file MPP-22-769-s009.docx]

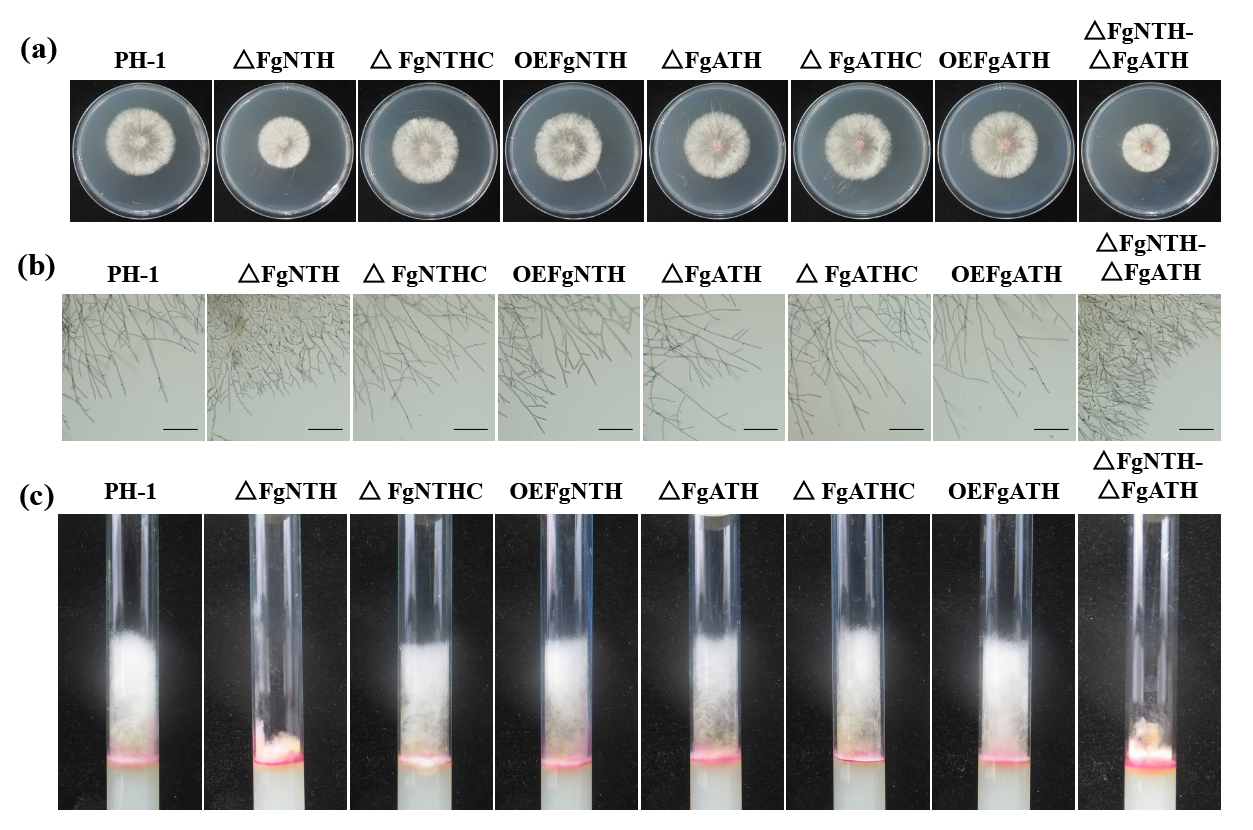


**Fig. S5** **Requirements of FgNTH for vegetative growth in *F. graminearum*.** **(a)** Hyphal penetration of each strain was determined on PDA medium plate with cellophane. Pictures were taken after cultured 3 days at 25ºC. **(b)** Branches at the tip of mycelium of each strain. All mutant strains and the wild type PH-1 were cultured on water agar medium plate with cellophane for 1 days at 25ºC, and then were observed using an Olympus IX-71 inverted fluorescence microscope (Tokyo, Japan). Bar = 200 μm. **(c)** The aerial hyphae of each strain. All mutant strains and the wild type PH-1 were cultured in sterilized glass tube containing PDA medium for 3 day at 25ºC.
