# Supplementary material for: Mechanism of validamycin A inhibiting DON biosynthesis and synergizing with DMI fungicides against Fusarium graminearum
Source: Mol Plant Pathol. 2021 May 2;22(7):769–85. doi: 10.1111/mpp.13060 (PMC8232029; doi:10.1111/mpp.13060)
Supplement: Supplementary file 6 [file MPP-22-769-s001.docx]

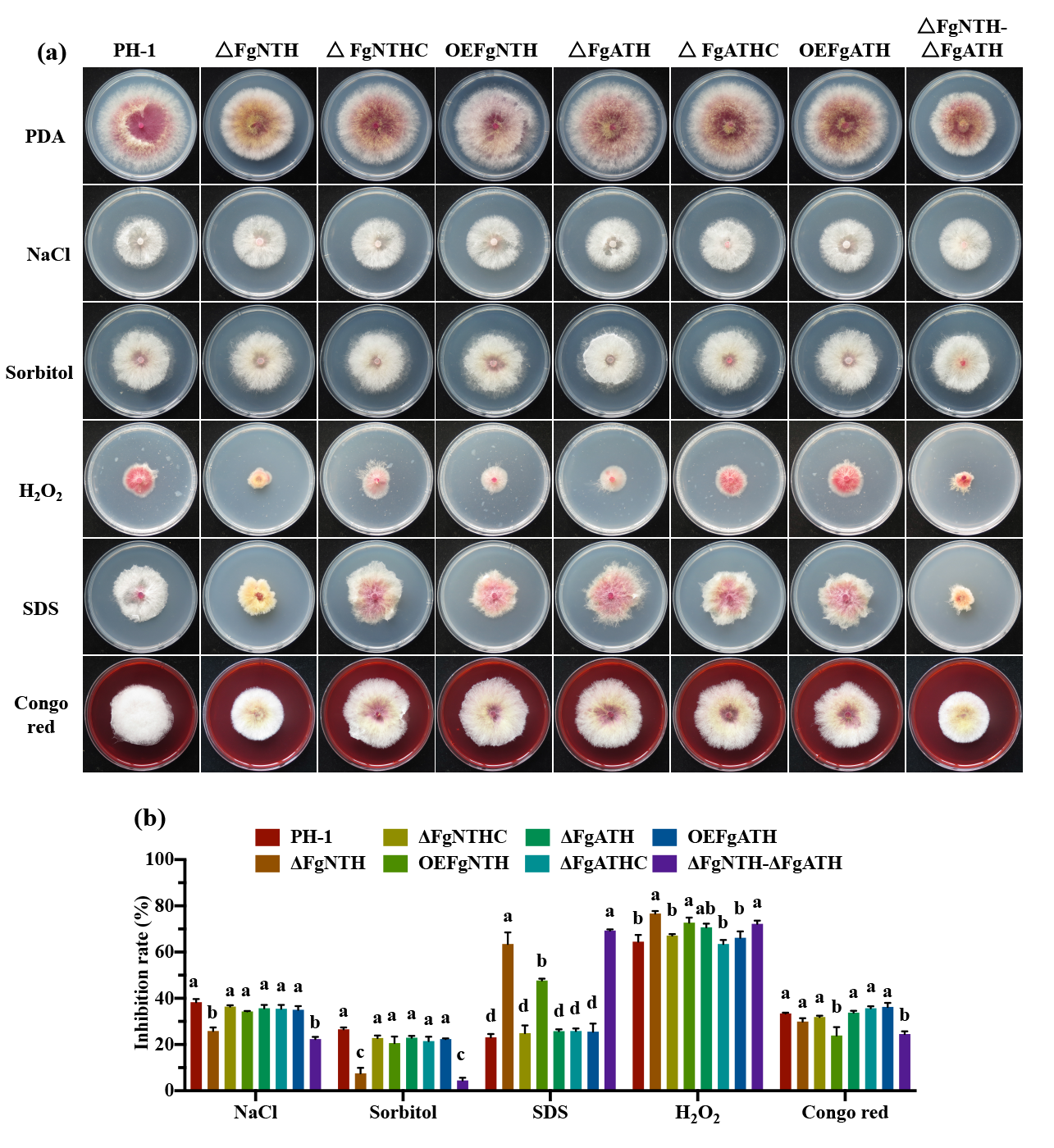


**Fig. S6** **Stress-sensitivity assays of the mutant strains of FgNTH and FgATH.** **(a)** Each strain was cultured on PDA medium for 2 days, and then transferred agar plugs (5 mm in diameter) containing mycelia of colony margin to PDA medium with 1.2 M NaCl, 1.2 M KCl, 1.2 M Sorbitol, 0.05% H_2_O_2_, 0.025%SDS and 0.05% Congo red for 3 days at 25ºC. Colony diameters were determined and pictures were taken after inoculated 5 days. **(b)** Percent inhibition of each strain under different stress. Each test was independently determined three times. The data were statistically analyzed using by one-way analyses of variance (ANOVA), and means were compared by the least significant difference at P < 0.05. The statistics and bar graphs were performed using GraphPad Prism 8.2.
