# Supplementary material for: Mechanism of validamycin A inhibiting DON biosynthesis and synergizing with DMI fungicides against Fusarium graminearum
Source: Mol Plant Pathol. 2021 May 2;22(7):769–85. doi: 10.1111/mpp.13060 (PMC8232029; doi:10.1111/mpp.13060)
Supplement: Supplementary file 7 [file MPP-22-769-s002.docx]

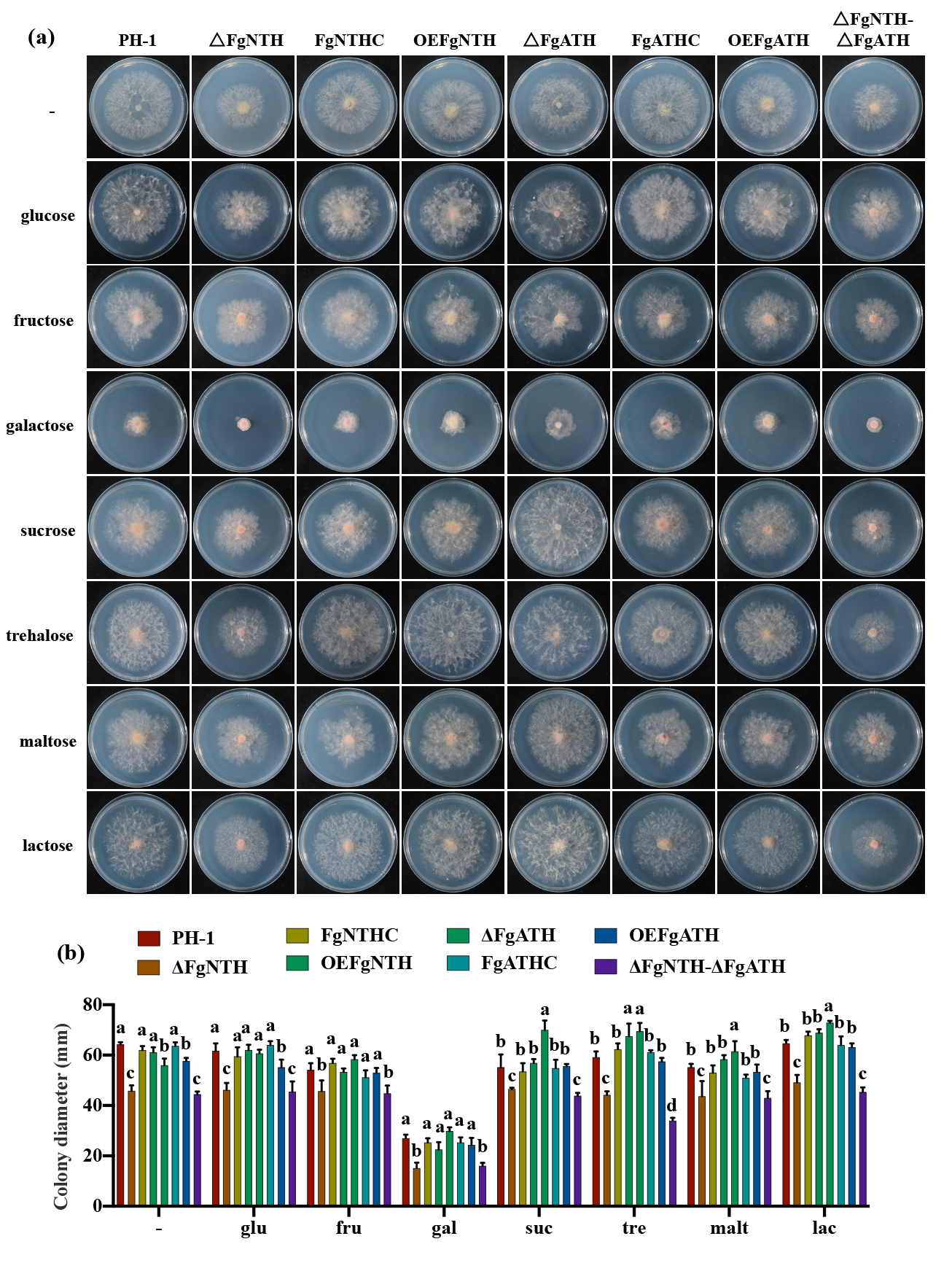


**Fig. S7** **FgNTH and FgATH affect the carbon resources utilization of *F. graminearum*. (a)** Each strain was cultured on PDA medium for 2 days, and then transferred agar plugs (5 mm in diameter) containing mycelia of colony margin to Czapek medium with 2% glucose, 2% fructose, 2% galactose, 2% sucrose, 2% trehalose, 2% maltose, and 2% lactose for 4-5 days at 25ºC. Colony diameters were determined and pictures were taken. **(b)** Mycelia growth of each strain under different carbon resources. Each test was independently determined three times. The data were statistically analyzed using by one-way analyses of variance (ANOVA), and means were compared by the least significant difference at P < 0.05. The statistics and bar graphs were performed using GraphPad Prism 8.2.
