# Supplementary material for: Mechanism of validamycin A inhibiting DON biosynthesis and synergizing with DMI fungicides against Fusarium graminearum
Source: Mol Plant Pathol. 2021 May 2;22(7):769–85. doi: 10.1111/mpp.13060 (PMC8232029; doi:10.1111/mpp.13060)
Supplement: Supplementary file 8 [file MPP-22-769-s012.docx]

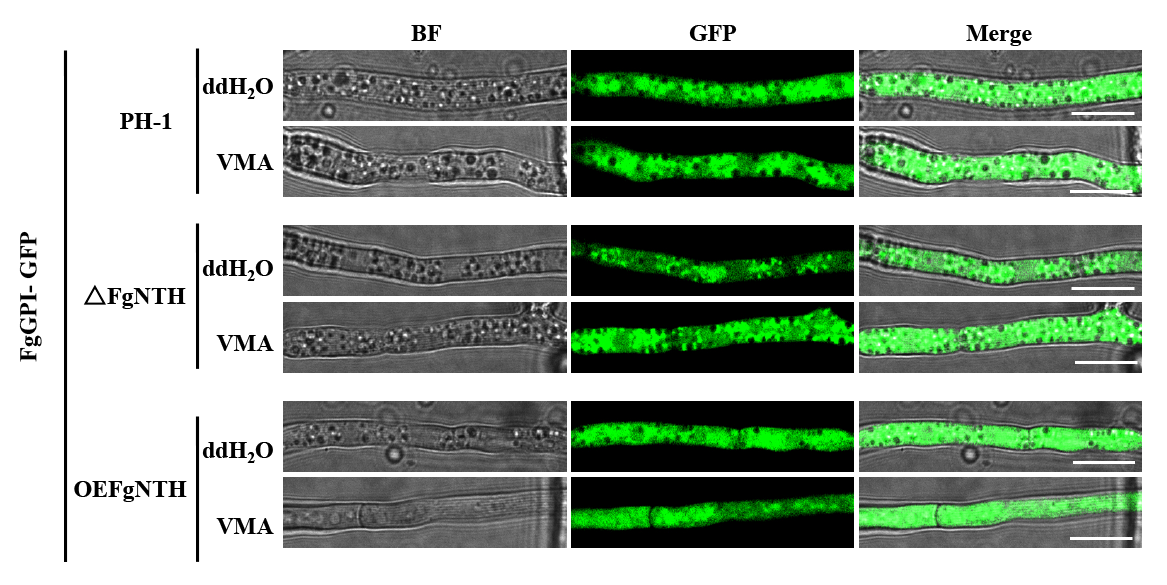


**Fig. S8 The fluorescence of FgGPI-GFP fusion protein in strains PH-1, △FgNTH-FgGPI-GFP and OEFgNTH- FgGPI-GFP with or without VMA treatment.** Fresh mycelia of strains FgGPI-GFP, △FgNTH-FgGPI-GFP and OEFgNTH- FgGPI-GFP were cultured in GYEP liquid medium with or without 10 μg mL^-1^ VMA for 3 days at 28ºC, and were harvested for observing fluorescence by using a Leica TCS SP5 confocal microscope (Wetzlar, Hessen, Germany). Bar = 10 μm.
