# Supplementary material for: Mechanism of validamycin A inhibiting DON biosynthesis and synergizing with DMI fungicides against Fusarium graminearum
Source: Mol Plant Pathol. 2021 May 2;22(7):769–85. doi: 10.1111/mpp.13060 (PMC8232029; doi:10.1111/mpp.13060)
Supplement: Supplementary file 9 [file MPP-22-769-s007.docx]

**Fig. S9** **The interaction between validamycin A and tebuconazole based on inhibition of mycelial growth of *F. graminearum*. (a) The** colony of the wild type strain PH-1 on Petri plates containing fungicides amended Czapek medium without carbon source. The concentration of validamycin A (VMA) is 10 μg mL^-1^ and the concentration of tebuconazole (TEB) is 0.05 μg mL^-1^. **(b)** Inhibition ration of VMA, TEB and the synergy between VMA and TEB. The data were statistically analyzed using by one-way analyses of variance (ANOVA), and means were compared by the least significant difference at P < 0.05. The statistics and bar graphs were performed using GraphPad Prism 8.2.
