# Supplementary material for: Mechanism of validamycin A inhibiting DON biosynthesis and synergizing with DMI fungicides against Fusarium graminearum
Source: Mol Plant Pathol. 2021 May 2;22(7):769–85. doi: 10.1111/mpp.13060 (PMC8232029; doi:10.1111/mpp.13060)
Supplement: Supplementary file 10 [file MPP-22-769-s006.docx]

Table S1. Wild-type and mutant strains of *F. graminearum* used in this study.

| Strain | Genotype description | Reference |
| --- | --- | --- |
| PH-1 | Wild-type strain of *F. graminearum* | This study |
| ∆FgNTH (∆FgNTH1) | *FgNTH* deletion mutant of PH-1 | This study |
| ∆FgATH (∆FgATH2) | *FgATH* deletion mutant of PH-1 | This study |
| ∆FgNTHC | Complement strain of ∆FgNTH | This study |
| ∆FgATHC | Complement strain of ∆FgATH | This study |
| ∆FgNTH-∆FgATH | *FgNTH* and *FgATH* double deletion mutant of PH-1 | This study |
| OEFgNTH (OEFgNTH3) | Overexpression strain of *FgNTH* | This study |
| OEFgATH (OEFgATH1) | Overexpression strain of *FgATH* | This study |
| FgNTH-GFP | *FgNTH*-GFP fusion strain of PH-1 | This study |
| FgATH-GFP | *FgATH*-GFP fusion strain of PH-1 | This study |
| FgPK-GFP | *FgPK*-GFP fusion strain of PH-1 | This study |
| FgGPI-GFP | *FgGPI*-GFP fusion strain of PH-1 | This study |
| FgTRI1-GFP | *FgTRI1*-GFP fusion strain of PH-1 | This study |
| FgTRI5-GFP | *FgTRI5*-GFP fusion strain of PH-1 | This study |
| ∆FgNTH-FgATH-GFP | *FgATH*-GFP fusion strain of ∆FgNTH | This study |
| ∆FgATH-FgNTH-GFP | *FgNTH*-GFP fusion strain of ∆FgATH | This study |
| ∆FgNTH-FgTRI1-GFP | *FgTRI1*-GFP fusion strain of ∆FgNTH | This study |
| ∆FgNTH-FgTRI5-GFP | *FgTRI5*-GFP fusion strain of ∆FgNTH | This study |
| ∆FgATH-FgTRI1-GFP | *FgTRI1*-GFP fusion strain of ∆FgATH | This study |
| ∆FgATH-FgTRI5-GFP | *FgTRI5*-GFP fusion strain of ∆FgATH | This study |
| FgNTH-3×flag | *FgNTH*- 3×flag fusion strain of PH-1 | This study |
| FgNTH-3×flag-FgPK-GFP | *FgPK*-GFP fusion strain of FgNTH-3×flag | This study |
| FgNTH-3×flag-FgGPI-GFP | *FgGPI*-GFP fusion strain of FgNTH-3×flag | This study |
| △FgNTH-FgPK-GFP | *FgPK*-GFP fusion strain of ∆FgNTH | This study |
| △FgNTH-FgGPI-GFP | *FgGPI*-GFP fusion strain of ∆FgNTH | This study |
| OEFgNTH-FgPK-GFP | *FgPK*-GFP fusion strain of OEFgNTH | This study |
| OEFgNTH-FgGPI-GFP | *FgGPI*-GFP fusion strain of OEFgNTH | This study |
| FgCYP51A-GFP | *FgCYP51A*-GFP fusion strain of PH-1 | This study |
| FgCYP51B-GFP | *FgCYP51B*-GFP fusion strain of PH-1 | This study |
| FgCYP51C-GFP | *FgCYP51C*-GFP fusion strain of PH-1 | This study |
| △FgNTH-FgCYP51A-GFP | *FgCYP51A*-GFP fusion strain of △FgNTH | This study |
| △FgNTH- FgCYP51B-GFP | *FgCYP51B*-GFP fusion strain of △FgNTH | This study |
| △FgNTH- FgCYP51C-GFP | *FgCYP51C*-GFP fusion strain of △FgNTH | This study |
| △FgATH-FgCYP51A-GFP | *FgCYP51A*-GFP fusion strain of △FgATH | This study |
| △FgATH- FgCYP51B-GFP | *FgCYP51B*-GFP fusion strain of △FgATH | This study |
| △FgATH- FgCYP51C-GFP | *FgCYP51C*-GFP fusion strain of △FgATH | This study |
| FgNTH-3×flag-FgCYP51A-GFP | *FgCYP51A*-GFP fusion strain of FgNTH-3×flag | This study |
| FgNTH-3×flag-FgCYP51B-GFP | *FgCYP51B*-GFP fusion strain of FgNTH-3×flag | This study |
| FgNTH-3×flag-FgCYP51C-GFP | *FgCYP51C*-GFP fusion strain of FgNTH-3×flag | This study |
