# Supplementary material for: Mechanism of validamycin A inhibiting DON biosynthesis and synergizing with DMI fungicides against Fusarium graminearum
Source: Mol Plant Pathol. 2021 May 2;22(7):769–85. doi: 10.1111/mpp.13060 (PMC8232029; doi:10.1111/mpp.13060)
Supplement: Supplementary file 11 [file MPP-22-769-s005.docx]

Table S2. Primers used in this study.

| Primer | Sequence (5’-3’) | Application |
| --- | --- | --- |
| FgNTH-upF (P1) | ATCGAGACGAAGACTTGAGAGG | Amplify *FgNTH* 5’ flank sequence, for gene knock out |
| FgNTH-upR (P2) | GCTCCTTCAATATCATCTTCTGTTGACAGCGTTGAAGTATCAGGC |  |
| FgNTH-dnF (P3) | GAAGGAGACAATACCGGAAGGAACGTTATTGCAGACTTGGAAGCGC | Amplify *FgNTH* 3’ flank sequence, for gene knock out |
| FgNTH-dnR (P4) | TATCCGCTCTGCGATCATATGC |  |
| FgATH-upF (P5) | ACGTATCGAGAATGAGAGTCGG | Amplify *FgATH* 5’ flank sequence, for gene knock out |
| FgATH-upR (P6) | GCTCCTTCAATATCATCTTCTGTTGAAGAGGAATGACCCAGTTGC |  |
| FgATH-dnF (P7) | GAAGGAGACAATACCGGAAGGAACAGAAGGCTGCGAAGAAGAGGTC | Amplify *FgATH* 3’ flank sequence, for gene knock out |
| FgATH-dnR (P8) | GAGCATGTCATCGAGGACAACG |  |
| HPH-Hsv F (P9) | ACAGAAGATGATATTGAAGG | Amplify HPH+Hsv-tk sequence |
| HPH-Hsv R (P10) | GTTCCTTCCGGTATTGTCTCCT |  |
| FgNTH-upyF (P11) | CGGAAGGTCATCTGACAGAGT | Transformants screen of △FgNTH |
| HPH-Hsv yR (P12) | CGTCCATCACAGTTTGCCAGT |  |
| HPH-Hsv yF (P13) | CCGAGACAATCGCGAACATCT | Transformants screen of △FgNTH |
| FgNTH-dnyR (P14) | CACCTCAATGGGTCACCTACT |  |
| FgATH-upyF (P15) | GGTTGGTGACACATTACCAGGT | Transformants screen of △FgATH |
| FgATH-dnyR (P16) | TCATGCTCACCAAGGGTGAGT | Transformants screen of △FgATH |
| NEO F (P17) | GTCGACAGAAGATGATATTG | Amplify NEO sequence |
| NEO R (P18) | TCAGAAGAACTCGTCAAGAAG |  |
| TrpC F (P19) | GTCGACAGAAGATGATATTGAAG | Amplify TrpC promoter sequence |
| TrpC R (P20) | ATCGATGCTTGGGTAGAATAGG |  |
| FgNTH yF (P21) | GAGGAAATCACCACTGATCTG | Amplify center fragment of *FgNTH* gene, for transformants screen |
| FgNTH yR (P22) | GACTGTTGAGATCAATGGTGG |  |
| FgATH yF (P23) | CAGATGATCAAGGCCTACGTC | Amplify center fragment of *FgATH* gene, for transformants screen |
| FgATH yR (P24) | GGGTTGTTCTTGAGGTAGTCG |  |
| FgATH F (P25) | ATGGCCGCGCCCTCTAATCAC | Amplify *FgNTH* gene |
| FgATH R (P26) | TGAACTAAGCAGACCAGCCAG |  |
| FgATH F (P27) | ATGCCGTCTCCACGACATATCG | Amplify *FgATH* gene |
| FgATH R (P28) | AAAGTAGAAGACACGACTCGAC |  |
| FgNTH pro F (P29) | GCACTGGTGGTCCATCTATG | Amplify *FgNTH* gene probe, for southern blot and transformants screen |
| FgNTH pro R (P30) | CCGTCGATATGTGACGCTGT |  |
| FgATH up-pro F  (P31) | CGGCATTGCCGATATCATGTTGC | Amplify *FgATH* gene up-stream probe, for southern blot and transformants screen |
| FgATH up-pro R (P32) | GACGATCGTTTCTTACCCACTGC |  |
| FgATH dn-pro F  (P33) | CTTCTCGACCCCTTCCTTGT | Amplify *FgATH* gene down-stream probe, for southern blot and transformants screen |
| FgATH dn-pro R (P34) | GTTGTAGTCCGCTCTCAGCT |  |
| FgATH NEO upR  (P35) | CAATATCATCTTCTGTCGACTGAAGAGGAATGACCCAGTTGC | Amplify *FgATH* 5’ flanksequence, for gene knock out in △FgNTH |
| FgATH NEO dnF (P36) | CTTCTTGACGAGTTCTTCTGAAGAAGGCTGCGAAGAAGAGGTC | Amplify *FgATH* 3’ flanksequence, for gene knock out in △FgNTH |
| FgATH NEO upyR  (P37) | CAGTCATAGCCGAATAGCCTCT | Transformants screen of △FgNTH-  △FgNTH |
| FgATH NEO dnyF  (P38) | CTGTCATCTCACCTTGCTCCT | Transformants screen of △FgNTH-  △FgNTH |
| FgNTH OE upR  (P39) | CTTCAATATCATCTTCTGTCGACGTTGACAGCGTTGAAGTATCAGG | Amplify *FgNTH* 5’ flanksequence, for constructing the trpC + *FgNTH* fragment |
| FgNTH OE dnF  (P40) | CCTATTCTACCCAAGCATCGATATGGCCGCGCCCTCTAATCACC | Amplify *FgNTH gene + FgNTH* 3’ flanksequence, for constructing the trpC-*FgNTH* fragment |
| FgATH OE upR  (P41) | CTTCAATATCATCTTCTGTCGACGATTGAAGAGGAATGACCCAGTTGC | Amplify *FgATH* 5’ flanksequence, for constructing the trpC + *FgATH* fragment |
| FgATH OE dnF  (P42) | CCTATTCTACCCAAGCATCGATATGCCGTCTCCACGACATATCG | Amplify *FgATH gene + FgATH* 3’ flanksequence, for constructing the trpC-*FgATH* fragment |
| FgNTH OE yF  (P43) | CTTGTTCTTGTCTCCTCCCT | Transformants screen of OEFgNTH |
| FgNTH OE yR  (P44) | CTCTCGGAGATTGACTCGCT |  |
| FgATH OE yF  (P45) | ACATGGTTGCCAACTATCGT | Transformants screen of OEFgATH |
| FgATH OE yR  (P46) | GAGAGGTTTCTCGAGCTTGT |  |
| FgNTH qF (P47) | CGTGCGAAAGTACAACCACG | *FgNTH* qRT-PCR analysis |
| FgNTH qR (P48) | GACTCCTTCGAGGCGGTATG |  |
| FgATH qF (P49) | GAAACGCCCAAGCTCCAAAG | *FgATH* qRT-PCR analysis |
| FgATH qR (P50) | GCCCTCGACAACTTCGTACT |  |
| FgActin qF (P51) | AGTACTCCGTCTGGATCGGT | Reference gene *FgActin* for qRT-PCR analysis |
| FgAtin qR (P52) | GATTGAAGGACCGCTCTCGT |  |
| FgPK qF (P53) | ACAACGGTGCTATCTGCTCC | *FgPK* qRT-PCR analysis |
| FgPK qR (P54) | CCGAACTTCAGATCGGCCTT |  |
| FgTRI1 qF (P55) | GAAGATTCCTGAAGGTCCCG | *FgTRI1* qRT-PCR analysis |
| FgTRI1 qR (P56) | TGTACCAATTCCAATCGCAGAC |  |
| FgTRI5 qF (P57) | GCGCATCGAGAATTTGCACT | *FgTRI5* qRT-PCR analysis |
| FgTRI5 qR (P58) | GCCTGTAGTCGCTTAGGGTC |  |
| FgTRI6 qF (P59) | CAGCTCAAGACCTGCAAGAA | *FgTRI6* qRT-PCR analysis |
| FgTRI6 qR (P60) | CCACCCTGCTAAAGACCCT |  |
| FgNTH-GFP F (P61) | TTTCGTAGGAACCCAATCTTCAAAATGGCCGCGCCCTCTAATCAC | For *FgNTH*-GFP fusion construct, RP27 promoter |
| FgNTH-GFP R (P62) | CACCACCCCGGTGAACAGCTCCTCGCCCTTGCTCACTGAACTAAGCAGACCAGCCAG |  |
| FgATH-GFP F (P63) | TTTCGTAGGAACCCAATCTTCAAAATGCCGTCTCCACGACATATC | For *FgATH*-GFP fusion construct, RP27 promoter |
| FgATH-GFP R (P64) | CACCACCCCGGTGAACAGCTCCTCGCCCTTGCTCACAAAGTAGAAGACACGACTCG |  |
| FgPK-GFP F (P65) | TTTCGTAGGAACCCAATCTTCAAAATGCCTCAGAAGTCAGAAAAC | For *FgPK*-GFP fusion construct, RP27 promoter |
| FgPK-GFP R (P66) | CACCACCCCGGTGAACAGCTCCTCGCCCTTGCTCACCTGAAGCTGGCCAATGCCGAG |  |
| FgGPI-GFP F (P67) | TTTCGTAGGAACCCAATCTTCAAAATGGCTCCCGCAAACACTCTC | For *FgGPI*-GFP fusion construct, RP27 promoter |
| FgGPI-GFP R (P68) | CACCACCCCGGTGAACAGCTCCTCGCCCTTGCTCACGTTCTGACCGTACTTCTTAAAGG |  |
| PYF11 yF (P69) | TTTCGTAGGAACCCAATCTTCAAA | PCR verification of GFP fusion construct |
| PYF11 yR (P70) | CACCACCCCGGTGAACAGCTCCTCGCCCTTGCTCAC |  |
| FgPK-AD F (P71) | TATGGCCATGGAGGCCAGTGATGCCTCAGAAGTCAGAAAAC | For constructing *FgPK*-pGADT7 AD fusion vector |
| FgPK-AD R (P72) | CAGCTCGAGCTCGATGGATCCTGAAGCTGGCCAATGCCGAG |  |
| FgPK-BD F (P73) | GCATATGGCCATGGAGGCCGATGCCTCAGAAGTCAGAAAAC | For constructing *FgPK*-pGBKT7 BD fusion vector |
| FgPK-BD R (P74) | CCGCTGCAGGTCGACGGATCCTGAAGCTGGCCAATGCCGAG |  |
| FgGPI-AD F (P75) | TATGGCCATGGAGGCCAGTGATGGCTCCCGCAAACACTCTC | For constructing *FgGPI*-pGADT7 AD fusion vector |
| FgGPI-AD R (P76) | CAGCTCGAGCTCGATGGATCGTTCTGACCGTACTTCTTAAAGG |  |
| FgGPI-BD F (P77) | GCATATGGCCATGGAGGCCGATGGCTCCCGCAAACACTCTC | For constructing *FgGPI*-pGBKT7 BD fusion vector |
| FgGPI-BD R (P78) | CCGCTGCAGGTCGACGGATCGTTCTGACCGTACTTCTTAAAGG |  |
| FgNTH-AD F (P79) | TATGGCCATGGAGGCCAGTGATGGCCGCGCCCTCTAATCAC | For constructing *FgNTH*-pGADT7 AD fusion vector |
| FgNTH-AD R (P80) | CAGCTCGAGCTCGATGGATCTGAACTAAGCAGACCAGCCAG |  |
| FgNTH-BD F (P81) | GCATATGGCCATGGAGGCCGATGGCCGCGCCCTCTAATCAC | For constructing *FgNTH*-pGBKT7 BD fusion vector |
| FgNTH-BD R (P82) | CCGCTGCAGGTCGACGGATCTGAACTAAGCAGACCAGCCAG |  |
| AD yF (P83) | CTATTCGATGATGAAGATACCCCACCAAACCC | PCR verification of pGADT7 AD fusion vector |
| AD yR (P84) | GTGAACTTGCGGGGTTTTTCAGTATCTACGATTC |  |
| BD yF (P85) | TCATCGGAAGAGAGTAGTAACAAAGG | PCR verification of pGBKT7 BD fusion vector |
| BD yR (P86) | CTAAGAGTCACTTTAAAATTTGTATAC |  |
| FgNTH-1×Flag up R (P87) | CTTGTCGTCGTCGTCCTTGTAGTCTGAACTAAGCAGACCAGCCAG | Amplify *FgATH* 5’ flanksequence+ *FgATH* gene *+* 1×Flag fragment |
| 2×Flag up R (P88) | CTTATCATCATCATCCTTGTAATCCTTGTCGTCGTCGTCCTTGTAGTC | Amplify *FgATH* 5’ flanksequence+ *FgATH* gene *+* 2×Flag fragment |
| 2×Flag dn F (P89) | GATTACAAGGATGATGATGATAAGGACTACAAGGACGACGACGACAAG | Amplify 2×Flag *+ FgATH* 3’ flanksequence fragment |
| FgNTH-3×Flag dn F (P90) | GACTACAAGGACGACGACGACAAGTAGGGTAATGTTATTGCAGAC | Amplify 3×Flag *+ FgATH* 3’ flanksequence fragment |
| FgNTH-3×Flag yF  (P91) | TGGCCTCCACAGCAAATGCT | Screening transformants of FgNTH-  3×Flag |
| FgNTH-3×Flag yR  (P92) | TACCTCTGTTGCCCTGACTC |  |
| FgCYP51A qF  (P93) | GCCAGCACTCTTCATCTTCT | *FgCYP51A* of qRT-PCR analysis |
| FgCYP51A qR  (P94) | CCTGGTAGAGTTCTTCGGTAATG |  |
| FgCYP51B qF  (P95) | ATCGTCCACCCGTTGTATTC | *FgCYP51B* of qRT-PCR analysis |
| FgCYP51B qR  (P96) | AGCAGTCGCCGTATTTATCTC |  |
| FgCYP51C qF  (P97) | CACCTTTATCCTCCTCGGAAAG | *FgCYP51C* of qRT-PCR analysis |
| FgCYP51C qR  (P98) | CTCAGCACACACATCCTTGA |  |
