# Supplementary material for: Mechanism of validamycin A inhibiting DON biosynthesis and synergizing with DMI fungicides against Fusarium graminearum
Source: Mol Plant Pathol. 2021 May 2;22(7):769–85. doi: 10.1111/mpp.13060 (PMC8232029; doi:10.1111/mpp.13060)
Supplement: Supplementary file 12 [file MPP-22-769-s011.docx]

**Table S3. A list of putative FgNTH-interacting proteins identified by affinity capture mass spectrometry assay.**

| Accession | Description |
| --- | --- |
| I1S821 | Mitoc_mL59 domain-containing protein |
| A0A2H3FYF2 | GTP-binding protein SAR1 |
| Q4I375 | Mitochondrial import inner membrane translocase subunit TIM16 |
| A0A2H3HNZ2 | Orotidine 5'-phosphate decarboxylase |
| **A0A1C3YL54** | **Pyruvate kinase** |
| Q4HZK7 | Protein BCP1 |
| A0A2H3H9I7 | Actin-related protein 2/3 complex subunit 4 |
| Q4IF76 | ATP-dependent RNA helicase DBP2 |
| I1RAH1 | Phosphotransferase |
| A0A2H3G2N0 | Mitogen-activated protein kinas |
| A0A2H3H1M0 | Histone H1/5 |
| I1RA32 | Peroxiredoxin |
| Q4HYR2 | Isocitrate lyase |
| A0A4U9ENX6 | 5-hydroxyisourate hydrolase |
| A0A2H3FEB8 | Cytochrome c1 |
| A0A2H3HJW2 | PKS_ER domain-containing protein |
| I1RSF6 | Mitochondrial pyruvate carrier |
| A0A2H3G7P0 | Chorismate synthase |
| A0A2H3HPP5 | Pyruvate carboxylase |
| A0A2H3G1J0 | Ubiquinone biosynthesis protein |
| A0A2H3HQZ1 | Protein MAK16 |
| V6R3L4 | Histone H4 |
| A0A1C3YLH1 | MFS domain-containing protein |
| Q4HZ95 | Mitochondrial import inner membrane translocase subunit TIM21 |
| I1RJK7 | UBIQUITIN_CONJUGAT_2 domain-containing protein |
| A0A2H3HB71 | Cell division control protein 42 |
| I1RR00 | Serine/threonine-protein kinase RIO1 |
| A0A2H3HD22 | GTP-binding protein ypt3 |
| Q4I1B1 | Vacuolar protein 8 |
| A0A2H3G8Q0 | PKS_ER domain-containing protein |
| A0A2H3FXV6 | Phosphoenolpyruvate carboxykinase |
| I1RWM4 | Malate synthase |
| A0A2H3HB64 | Multifunctional tryptophan |
| A0A2H3H8D5 | Bifunctional cytochrome P450/NADPH--P450 reductase |
| A0A4U9EN16 | Glycerol-3-phosphate dehydrogenase |
| A0A2H3GV25 | Mannan polymerase II complex ANP1 subunit |
| I1RJL9 | Dolichol-phosphate mannosyltransferase subunit 1 |
| A0A2H3GF34 | GTP cyclohydrolase-2 |
| A0A2H3GAC3 | FAD-binding FR-type domain-containing protein |
| A0A2H3H1M6 | Cytochrome b5 heme-binding domain-containing protein |
| A0A2H3GKK5 | Mannose-6-phosphate isomerase |
| A0A2H3GMK9 | Mannitol-1-phosphate dehydrogenase |
| **B9UZE9** | **Cytochrome P450 51B** |
| A0A2H3FXD0 | Mitotic control protein dis3 |
| A0A4U9F7M3 | Threonine dehydratase |
| A0A2H3HCB8 | Myosin-2B |
| A0A2H3G032 | MAP kinase kinase |
| A0A2H3FRB4 | Isocitrate dehydrogenase [NAD] subunit, mitochondrial |
| I1RM44 | Glycerol-3-phosphate dehydrogenase [NAD(+)] |
| I1RBY9 | Acetolactate synthase |
| A0A2H3H225 | Tryptophan synthase |
| A0A2H3HU66 | Msp1 |
| I1RWB4 | Rab proteins geranylgeranyltransferase |
| A0A4U9F4E8 | Prephenate dehydrogenase [NADP(+)] |
| A0A2H3H9B5 | Vacuolar protein sorting-associated protein 4 |
| V6R3U5 | Calcium-transporting ATPase |
| A0A2H3GN39 | Proteasome subunit alpha type |
| A0A2H3FW58 | PKS_ER domain-containing protein |
| A0A4U9EMX9 | Fe2OG dioxygenase domain-containing protein |
| A0A2H3FT63 | NADH-cytochrome b5 reductase |
| A0A2H3FW98 | DLH domain-containing protein |
| Q8TG19 | Mitogen-activated protein kinase |
| A0A2H3G214 | Methylenetetrahydrofolate dehydrogenase |
| V6RDH3 | Cystathionine beta-synthase |
| A0A1C3YLI1 | Methylenetetrahydrofolate reductase |
| A0A2H3FG69 | Acetolactate synthase small subunit |
| A0A2H3GP30 | Beta-lactamase domain-containing protein |
| A0A2H3HBF5 | STE/STE11 protein kinase |
| I1R9Y2 | Alkyl transferase |
| A0A2H3HGW6 | Dihydroxy-acid dehydratase |
| A0A2H3G0I7 | Cell division control protein 2 |
| A0A2H3FUN1 | Protein phosphatase 1 regulatory subunit SDS22 |
| I1S2E9 | Ubiquitin-like domain-containing protein |
| A0A2H3G3G5 | GPI-anchor transamidase |
| Q4I8P5 | Cytochrome c oxidase assembly protein COX16, mitochondrial |
| A0A2H3GEU2 | Zinc-finger protein ZPR1 |
| A0A2H3FCN6 | Vacuolar protein sorting-associated protein 17 |
| A0A2H3HN25 | Citrate synthase |
| I1RWD1 | Phospholipid-transporting ATPase |
| A0A2H3H888 | Heat shock protein 90 |
| A0A2H3GCM1 | Quinate permease |
| Q0Q0M5 | RBR-type E3 ubiquitin transferase |
| A0A2H3FRT9 | Flavodoxin-like domain-containing protein |
| A0A2H3HB05 | Lysophospholipase NTE1 |
| Q876W1 | Monooxygenase |
| A0A2H3FU73 | Pyruvate decarboxylase |
| V6RQW4 | Imidazole glycerol phosphate synthase hisHF |
| A0A2H3GQB9 | Mitochondrial presequence protease |
| I1RQH3 | Eukaryotic translation initiation factor 3 subunit K |
| Q4I5I4 | Transcription elongation factor SPT5 |
| A0A4U9ET23 | Kinesin-like protein |
| A0A2H3FDM1 | Histone H2B |
| I1RZD0 | Phosphomevalonate kinase |
| A0A2H3FUP6 | Serine/threonine-protein phosphatase |
| I1RA81 | MAP kinase kinase kinase |
| A0A1C3YIF8 | Myosin motor domain-containing protein |
| A0A2H3HD06 | Glutaryl-CoA dehydrogenase |
| A0A2H3G1T1 | Heat shock protein SSC1 |
| A0A2H3G780 | Myosin-5 |
| A0A2H3FL45 | Glyceraldehyde-3-phosphate dehydrogenase |
| A0A2H3G0U0 | Tubulin beta chain |
