# Supplementary material for: Mechanism of validamycin A inhibiting DON biosynthesis and synergizing with DMI fungicides against Fusarium graminearum
Source: Mol Plant Pathol. 2021 May 2;22(7):769–85. doi: 10.1111/mpp.13060 (PMC8232029; doi:10.1111/mpp.13060)
Supplement: Supplementary file 13 [file MPP-22-769-s003.docx]

Table S4. Concentration gradients used to determine the sensitivity of *F. graminearum* to validamycin A and tebuconazole and in mixtures*

| **Fugicide treatment** | **Concentration gradient (μg/mL)** | | | | |
| --- | --- | --- | --- | --- | --- |
| Validamycin A (VMA) | 0.1 | 1 | 10 | 100 |  |
| Tebuconazole (TEB) | 0.01 | 0.04 | 0.16 | 0.64 | 2.56 |
| VMA:TEB (1: 1) | 0.01 | 0.04 | 0.16 | 0.64 | 2.56 |
| VMA:TEB (1: 2) | 0.01 | 0.04 | 0.16 | 0.64 | 2.56 |
| VMA:TEB (2: 1) | 0.01 | 0.04 | 0.16 | 0.64 | 2.56 |
| VMA:TEB (1: 3) | 0.01 | 0.04 | 0.16 | 0.64 | 2.56 |
| VMA:TEB (3: 1) | 0.01 | 0.04 | 0.16 | 0.64 | 2.56 |

*Sensitivity was determined based on mycelia growth on Petri plates containing fungicide- amended Czapek medium without carbon source. Each value in the table indicates the total concentration of fungicide in the medium.
