# Supplementary material for: Mechanism of validamycin A inhibiting DON biosynthesis and synergizing with DMI fungicides against Fusarium graminearum
Source: Mol Plant Pathol. 2021 May 2;22(7):769–85. doi: 10.1111/mpp.13060 (PMC8232029; doi:10.1111/mpp.13060)
Supplement: Supplementary file 14 [file MPP-22-769-s008.docx]

Table S5. Inhibition ration of validamycin A on *F. graminearum* on Czapek medium without carbon source.

| Validamycin A  (μg/mL) | 0.1 | 1 | 10 | 100 |
| --- | --- | --- | --- | --- |
| Inhibition ration (%) | 40.77 | 43.84 | 47.44 | 43.06 |
