# Supplementary material for: Mechanism of validamycin A inhibiting DON biosynthesis and synergizing with DMI fungicides against Fusarium graminearum
Source: Mol Plant Pathol. 2021 May 2;22(7):769–85. doi: 10.1111/mpp.13060 (PMC8232029; doi:10.1111/mpp.13060)
Supplement: Supplementary file 15 [file MPP-22-769-s010.docx]

Table S6. Interactions between validamycin A and tebuconazole based on inhibition of mycelial growth of *F. graminearum*.

| **Fungicides** | **Linear regression equation** | **R^2^** | **Observed EC_50_ (μg/mL)** | **Expected EC_50_ (μg/mL)** | **Synergy ratio* (SR)** |
| --- | --- | --- | --- | --- | --- |
| Validamycin A (VMA) | - | - | ~10 | - |  |
| Tebuconazole (TEB) | Y=6.278+0.7519X | 0.9995 | 0.0200 | - |  |
| VMA:TEB (1: 1) | Y=6.5094+0.9553X | 0.9481 | 0.0263 | 0.0399 | 1.5179 |
| VMA:TEB (1: 2) | Y=6.7705+0.9925X | 0.9964 | 0.0165 | 0.0300 | 1.8164 |
| VMA:TEB (2: 1) | Y=6.4902+0.9200X | 0.993 | 0.0240 | 0.0598 | 2.4900 |
| VMA:TEB (1: 3) | Y=7.0074+1.0817X | 0.9823 | 0.0139 | 0.0266 | 1.9172 |
| VMA:TEB (3: 1) | Y=6.3182+0.8755X | 0.9983 | 0.0312 | 0.0795 | 2.5488 |

*Ration between expected and observed EC_50_ values.
